# VirHunter: A Deep Learning-Based Method for Detection of Novel RNA Viruses in Plant Sequencing Data

**DOI:** 10.3389/fbinf.2022.867111

**Published:** 2022-05-13

**Authors:** Grigorii Sukhorukov, Maryam Khalili, Olivier Gascuel, Thierry Candresse, Armelle Marais-Colombel, Macha Nikolski

**Affiliations:** ^1^ CNRS, IBGC, UMR 5095, Université de Bordeaux, Bordeaux, France; ^2^ Bordeaux Bioinformatics Center, Université de Bordeaux, Bordeaux, France; ^3^ Université de Bordeaux, INRAE, UMR BFP, CS20032, CEDEX, Villenave d’Ornon, France; ^4^ Institut de Systématique, Biodiversité, Evolution (ISYEB - UMR7205, Muséum National d’Histoire Naturelle, CNRS, SU, EPHE, UA), Paris, France

**Keywords:** novel virus detection, RNA viruses, plant virome, alignment-free method, deep learning, artificial neural network

## Abstract

High-throughput sequencing has provided the capacity of broad virus detection for both known and unknown viruses in a variety of hosts and habitats. It has been successfully applied for novel virus discovery in many agricultural crops, leading to the current drive to apply this technology routinely for plant health diagnostics. For this, efficient and precise methods for sequencing-based virus detection and discovery are essential. However, both existing alignment-based methods relying on reference databases and even more recent machine learning approaches are not efficient enough in detecting unknown viruses in RNAseq datasets of plant viromes. We present VirHunter, a deep learning convolutional neural network approach, to detect novel and known viruses in assemblies of sequencing datasets. While our method is generally applicable to a variety of viruses, here, we trained and evaluated it specifically for RNA viruses by reinforcing the coding sequences’ content in the training dataset. Trained on the NCBI plant viruses data for three different host species (peach, grapevine, and sugar beet), VirHunter outperformed the state-of-the-art method, DeepVirFinder, for the detection of novel viruses, both in the synthetic leave-out setting and on the 12 newly acquired RNAseq datasets. Compared with the traditional tBLASTx approach, VirHunter has consistently exhibited better results in the majority of leave-out experiments. In conclusion, we have shown that VirHunter can be used to streamline the analyses of plant HTS-acquired viromes and is particularly well suited for the detection of novel viral contigs, in RNAseq datasets.

## Introduction

Study of viromes at an unprecedented scale has been enabled by the adoption of high-throughput sequencing (HTS) technologies and is now frequently undertaken across an increasing range of host species. In particular, sequencing of plant viromes has become quite common, partly due to its relevance to the agricultural sector. The acquired datasets help to elucidate important questions such as virus spread among host reservoirs and effects of agriculture on the ecosystems and their biodiversity as well as the identification of novel viruses in crops and natural environments (Lefeuvre et al., 2019). These developments are fast advancing our knowledge of viral diversity through the discovery of previously unknown viral species or variants and the identification of new hosts of known viruses (Roossinck et al., 2015; Massart et al., 2017). Following the classification proposed by Stobbe and Roossinck (2014), viruses identified in HTS datasets can be classified into three different groups as follows: 1) viruses that are already known to infect a given host; 2) novel viruses from a known family or known viruses that have not been found previously described to infect a given host; and finally 3) completely novel viruses that share little to no sequence similarity with known viruses already present in the databases.

Using an efficient virus detection method, including for the identification of novel viruses, is essential for efficient disease management. Standard diagnostic tests (ELISA assays and PCR-based assays) depend on specific antibodies or primers and thus require prior knowledge of the virus and of its phylogenetic neighbors. Precise identification of viruses is further complexified by the large diversity encountered in the majority of viral species which is linked to the high mutation rate of these agents. This is particularly true for plant viruses, the majority of which are RNA viruses whose mutation rate is very high (Jenkins et al., 2002). Moreover, the new variants emerging from genomic rearrangements or recombination events can also significantly differ from the parental viruses (Domingo 2010). Also, many of the plant viruses are multihost pathogens, and a single plant can be infected by multiple unrelated viral species (Roossinck, 1997). Such infections by multiple viruses represent an additional challenge for detection since the viral load of different pathogens can be very unequal (Martín and Elena, 2009). Moreover, in most cases, background contamination is currently unavoidable (Kleiner et al., 2015; Maree et al., 2018; Kutnjak et al., 2021). In this context, HTS combined with bioinformatics tools has been shown to be a valuable approach, both for detection of known viruses and for the discovery of novel ones (Maree et al., 2018; Villamor et al., 2019; Mehetre et al., 2021).

Viruses do not have a universal gene marker that could be used for their identification, contrary to the conserved regions of the 16S rRNA and ITS genes, commonly used to classify bacteria and fungi at the genus or species level (Mokili et al., 2012). Moreover, the abundance of viral genomic material in plant sequencing samples can be very low (Massart et al., 2019), due to the dominance of the host material. Hence, specific sample preparation to enrich plant RNA viral-specific sequences is an important step that makes the downstream detection of viruses by bioinformatics methods more reliable. They include approaches providing a high and targeted enrichment of viral sequences, such as the purification of viral double-stranded RNAs (dsRNAs) or that of virion-associated nucleic acids (VANAs) as well as less specific approaches generally affording lower enrichment, such as the sequencing of small interfering RNAs (siRNAs) or inclusion of a ribodepletion step prior to the sequencing of total cellular RNAs (Maree et al., 2018; Kutnjak et al., 2021). As already discussed in a range of reviews, each of these approaches have advantages and weaknesses. In particular, strategies providing high enrichment factors may improve detection sensitivity but often at the cost of introducing biases with the risk of compromising the detection of some particular viruses (Maree et al., 2018; Kutnjak et al., 2021). For example, dsRNA-based approaches are usually poor at detecting DNA viruses, while VANA-based ones may perform poorly for viruses with labile particles.

When interested in known viruses or potentially novel viruses but from a known family, bioinformatics methods that compare the sequenced reads to genomes in public databases are very efficient for virus detection and identification (Stobbe and Roossinck, 2014; Massart et al., 2019). Read-based analysis is thus particularly suited to study viral diversity of sequencing samples in terms of known viral species. Generalistic metagenome analysis tools such as, for example, Kaju (Menzel et al., 2016), Kraken 2 (Wood et al., 2019), and Centrifuge (Kim et al., 2016) show good performance in terms of sensitivity and precision in detection of present known viral species (De Vries et al., 2021).

For the discovery of novel viruses, use of *de novo* assembly to recover novel viral contigs from sequencing data is an essential step in order to overcome the incompleteness of virus reference databases, annotation errors and, importantly, the limited homology between novel viral sequences and reference genomes (Sutton et al., 2019). The assembly step is a staple of short-read sequencing studies, which are still the vast majority today (Maree et al., 2018; Kutnjak et al., 2021). It represents its own challenges, in particular, for very short reads such as those of siRNAs and for viral populations with multiple and microdiverse variants (Warwick-Dugdale et al., 2019), often leading to microdiversity-associated fragmentation and, sometimes, to chimeras in the resulting contigs (Martinez-Hernandez et al., 2017; Roux et al., 2017), which in turn affects the downstream analysis, including estimation of viral diversity and identification of novel viruses (Nayfach et al., 2021). Popular assembler choices are the generalistic de Bruijn graph assembly metaSPAdes (Nurk et al., 2017) and Trinity, for RNAseq (Grabherr et al., 2011).

Following the recent review (Kutnjak et al., 2021), the methods used to analyze assembled contigs can be grouped into three main categories: 1) alignment and mapping-based methods, 2) protein domain searches, and 3) k-mer-based approaches that can either rely on signatures or leverage machine learning. Among this large plethora of tools, alignment-based methods are widely adopted when working with assembled contigs since they provide a longer sequence for homology search against reference genomes using either BLAST (Altschul et al., 1990) and its derivatives or the amino acid alignment of protein-coding genes predicted from the assembled contigs using DIAMOND (Buchfink et al., 2015). Also, focusing the analysis on coding regions is particularly relevant for RNAseq data since the non-coding sequences of viruses are not highly represented in such samples, even if they can be well conserved in certain viral taxa. However, the main drawback of alignment- or mapping-based approaches lies on the fact that they are both computationally intensive and require expertise for filtering and interpreting the results. As for the generalistic k-mer signature approaches, they remain demanding in terms of memory and are best suited for diversity analysis tasks (Kutnjak et al., 2021).

The emergence of machine learning tools for contig-based analysis of virome sequencing data holds much promise to streamline the discovery of novel viruses in sequencing datasets by both avoiding the time-consuming sequence similarity analyses and modeling even highly divergent sequences. These methods build models based on sequences with known class labels such as “virus” and “host” and learn features that allow them to differentiate between the classes. VirFinder (Ren et al., 2017) and VirSorter2 (Guo et al., 2021) rely on classical machine learning, the former being based on a regularized logistic regression applied to the k-mer frequency matrix extracted from the sequence and the latter on a random forest model built from genomic features. Methods based on deep learning networks have also been proposed for virus detection, such as DeepVirFinder (Ren et al., 2020) and ViraMiner (Tampuu et al., 2019) that both rely on a combination of convolutional neural networks (CNNs) and dense neural networks, and VirNet (Abdelkareem et al., 2018) that relies on a long short-term memory (LSTM) architecture. These three deep learning methods were developed for identification of viral contigs in metagenomic samples and evaluated on bacterial and human metagenomes. However, DeepVirFinder has been recently successfully used in plant-related virome studies (Santos-Medellin et al., 2021).

In this work, we present VirHunter, a deep learning method that uses convolutional neural networks (CNNs), classifies previously assembled contigs to identify potential viral, host, and bacterial (contamination) sequences in RNAseq samples. The hybrid architecture of VirHunter combines a multi-network CNN-based module covering different k-mer sizes with a downstream random forest classifier module. We have trained VirHunter models for three different plant host species: peach, grapevine, and sugar beet. Importantly, we have shown that VirHunter is especially performant for the task of completely novel virus discovery by building 31 leave-out datasets, in which each viral family is excluded from the training dataset, and comparing the results with a standard BLAST-based solution on one side and a state-of-the-art deep learning method, DeepVirFinder, the other side. VirHunter not only systematically outperformed DeepVirFinder in terms of virus detection but also has considerably reduced the False Positive rate. Cross-evaluation has shown that host detection accuracy remained high and decreased slightly when test sequences originated from the plant species were further phylogenetically removed from that used to train the model. We have further evaluated the detection capacity of VirHunter on *in silico* mutated contigs sampled from the NCBI virus database and have shown that it decreased only slightly with a progressively increased mutation rate (e.g., True Positive rate of 0.898 for 20% mutation rate). Moreover, we generated 12 RNAseq datasets for a range of host species and have shown that VirHunter was not only able to uncover the viruses that were previously identified but also to streamline the analyses by considerably reducing the need for manual curation.

## Materials and Methods

### Datasets

We downloaded all complete and incomplete viral sequences from the NCBI virus database for which the host’s taxonomic id belongs to *Viridiplantae* on 26/10/2021, which yielded 122,832 sequences. Plant sequences have been downloaded for *Prunus persica* (peach), *Vitis vinifera* (grapevine), *Beta vulgaris* (sugar beet), and *Oryza sativa* (rice) from the NCBI RefSeq genomes database. On one hand, they consist of the latest available assemblies, GCF_000346465.2, GCF_000003745.3, GCF_000511025.2, and GCF_001433935.1 for peach, grapevine, sugar beet, and rice, respectively, and of the coding region sequences (CDSs), on the other hand. In the absence of the plastid sequence in the reference assembly of the sugar beet, we used the separately available sugar beet plastid sequence (NC_059012.1). All complete representative bacterial genomes have been downloaded from the NCBI RefSeq database on 28/10/2021 using the genome_updater.sh script.

To simulate the discovery of completely unknown viruses that do not have expected similarities with the available data, we constructed virus family leave-out datasets by excluding in turns all the sequences of a given plant viral family from the downloaded virus dataset. The NCBI taxonomy contains 45 viral families. We excluded the *Pospiviroidae* and the *Avsunviroidae* families of viroids as they have very small genomes (average length < 1,000). All families with the number of available sequences <100 were merged in one dataset called *small families*. Finally, all sequences without family labels constituted the *unclassified* dataset. This resulted in 31 leave-out datasets.

Moreover, we generated 12 novel virome-sequencing RNAseq datasets, sampled from peach, grapevine, and sugar beet (see *Sample Preparation and Sequencing*). Description of these datasets and presence of viruses identified by aligning assembled contigs against the NCBI GenBank database (see *Assembly of RNAseq Datasets and Annotation of Viral Contigs*) are listed in the Supplementary Table S1.

### Sample Preparation and Sequencing

Total RNAs were extracted from three peach leaf samples, three grapevine phloem scrapping samples, and three sugar beet leaf samples using the CTAB method (Chang et al., 1993), the Spectrum™ Plant Total RNA Kit (Sigma-Aldrich, Saint Quentin-Fallavier, France), and the NucleoSpin RNA plant kit (Macherey-Nagel SAS, Hoerdt, France), respectively. RNAseq libraries were prepared either from total RNAs (peach and grapevine samples), messenger RNAs (grapevine samples), or ribodepleted RNAs (sugar beet samples). High-throughput sequencing was performed on an Illumina platform (Hiseq3000 or NovaSeq600) using a paired-end read length of 2 × 150 bp. Accession numbers for each of the three studies (peach, grapevine, and sugar beet) containing raw FASTQ sequencing files are provided in the Supplementary Table S1.

### Assembly of RNAseq Datasets and Annotation of Viral Contigs

All of the 12 selected plant virome datasets (see *Datasets*) were processed with the QIAGEN CLC Genomics Workbench (v.21.0.5). Briefly, reads were first quality-controlled and trimmed using default parameters and then assembled using *de novo* assembly (word size 50, bubble size 300, and minimum contig length 250). To identify viral contigs present in these datasets, we followed a standard three step BLAST-based approach, see e.g., (Candresse et al., 2018). 1) All contigs were aligned using the CLC built in tBLASTx tool against the NCBI nucleotide non-redundant database limited to taxonomic identifiers of viruses. Contigs having significant hits (e-value below the $10^{-20}$ cut off) were selected. 2) Contigs were further filtered by aligning them using BLASTn and BLASTx with default parameters against the whole GenBank non-redundant nucleotide and protein databases, respectively, and keeping contigs for which the best hits correspond to plant viruses for both BLASTn and BLASTx. Additional manual expert curation allowed to discard contigs with incoherencies between the two alignment results. 3) Finally, all reads passing quality control were mapped against the plant viral contigs, resulting from step 2 using the CLC built-in mapping utility with default parameters with high stringency (90% identity of 90% of read’s length). Only contigs with length >750 nucleotides and having sufficient read coverage (expert curation) were retained.

Annotation results together with the counts of thus identified viral contigs are listed in the Supplementary Table S1.

### Data Preprocessing

**FIGURE 1:**
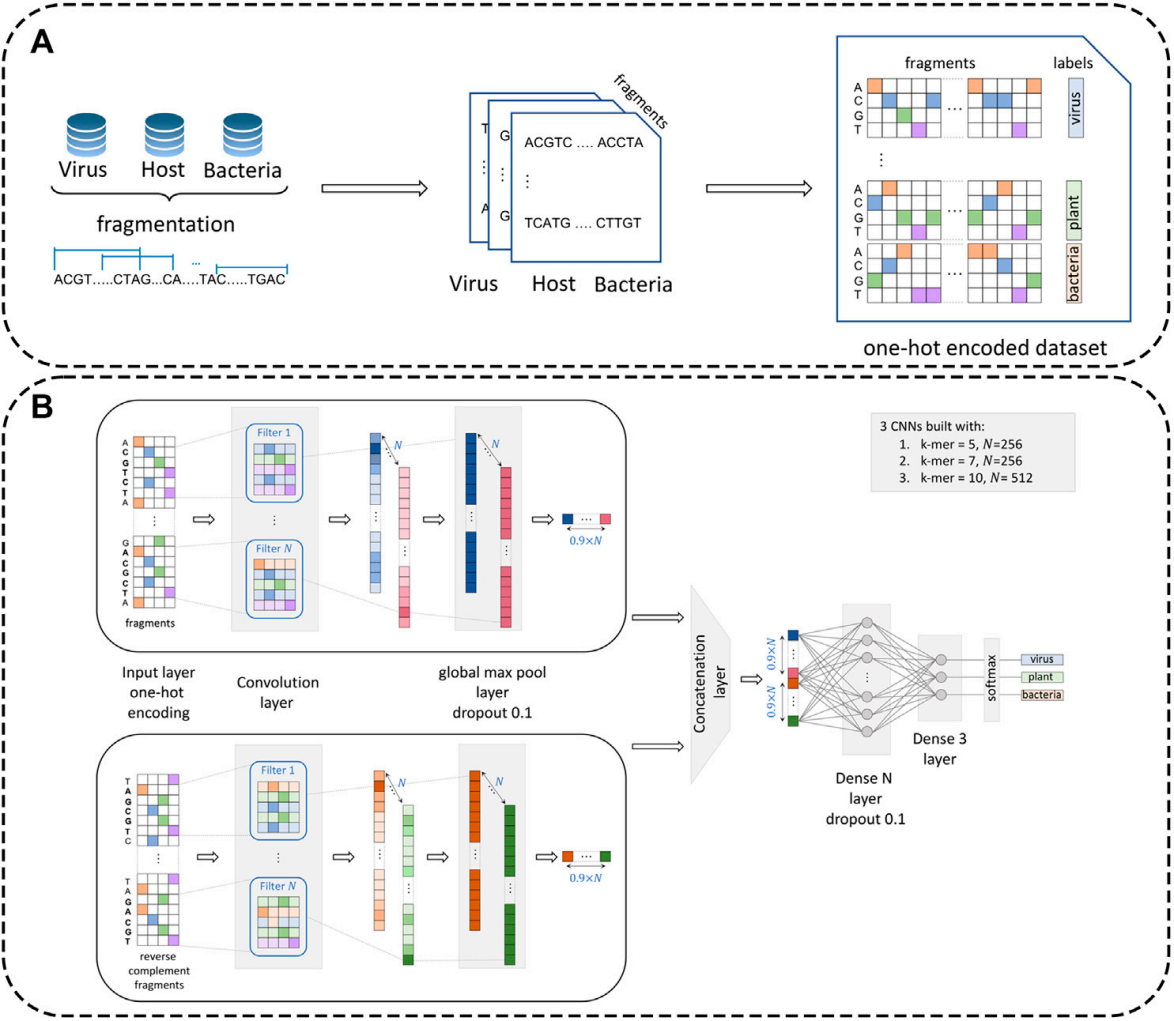
Dataset preprocessing procedure and architecture of the multi-CNN module. Panel **(A)**: Reference datasets (virus, plant, and bacteria) are first fragmented with a pre-defined fragment size ( 500 and 1000 bp). Each fragment is further one-hot encoded and carries the class label. Panel **(B)**: Three CNN modules are built for k-mers of size k=5,7 , and 10 . One-hot encoded genomic fragments of a fixed size are processed by convolutional and global max-pooling layers before being concatenated. A total of two dense layers are followed by the softmax activation function to produce a 3-class classification.

To prepare the data for processing by the neural network module, datasets were preprocessed by creating representative one-hot encoded fragments (see Figure 1). Specifically, let us denote the virus dataset by V , the plant dataset by H (for “host”)—composed of the full assembly G , the coding sequences C , the chloroplast sequence L , and the bacterial dataset by B . Given a fragment size n  of 500 and 1,000 nucleotides, V was split into fragments of size n with a sliding window with an increment of n/2 . Sequences shorter than n nucleotides and longer than 0.95×n  were padded to n bp length with gaps (those shorter than 0.95×n  are discarded), together yielding N viral fragments. Same number *N* of fragments of size n was randomly sampled from B. As for the plant, G was split into 0.6×N fragments using a sliding window with an increment of size n , C was split into 0.3×N fragments with a sliding window with increment of n/2 , and finally 0.1×N fragments were sampled randomly from L .

Including plastids in relatively high proportion into the plant dataset H  was important to avoid the potential incorrect assignment of contigs originating from plastids to B , given the phylogenetic proximity of plastids and bacteria (McFadden, 2001). Moreover, there are RNA viruses that are known to be replicated in tight association with plastids (mostly chloroplasts) - see e.g., (Budziszewska and Obrępalska-Stęplowska, 2018; Delgado et al., 2019). Enriching for CDS sequences was necessary since the envisioned application of VirHunter is for RNAseq virome datasets. Five compositions of G/C/L proportions of H were tested (100/0/0, 90/0/10, 60/30/10, 50/40/10, and 45/45/10, data not shown) and the best was retained.

Fragments were further transformed from length n ACGT-character strings to n×4 one-hot encoded arrays, in which an A is encoded by [1, 0, 0, 0], a C is encoded by [0, 1, 0, 0] *etc*., while gaps are encoded by [0, 0, 0, 0]. Moreover, the encoded dataset is augmented by adding the reverse complement of each original fragment. Indeed, it has been shown by Shrikumar et al. (2017) that CNN models in genomics require the reverse-complement data augmentation combined with parameter sharing between the forward- and reverse-complement representations of the model. Class labels V, H , or B are assigned to each fragment according to its provenance.

### VirHunter Architecture

**FIGURE 2:**
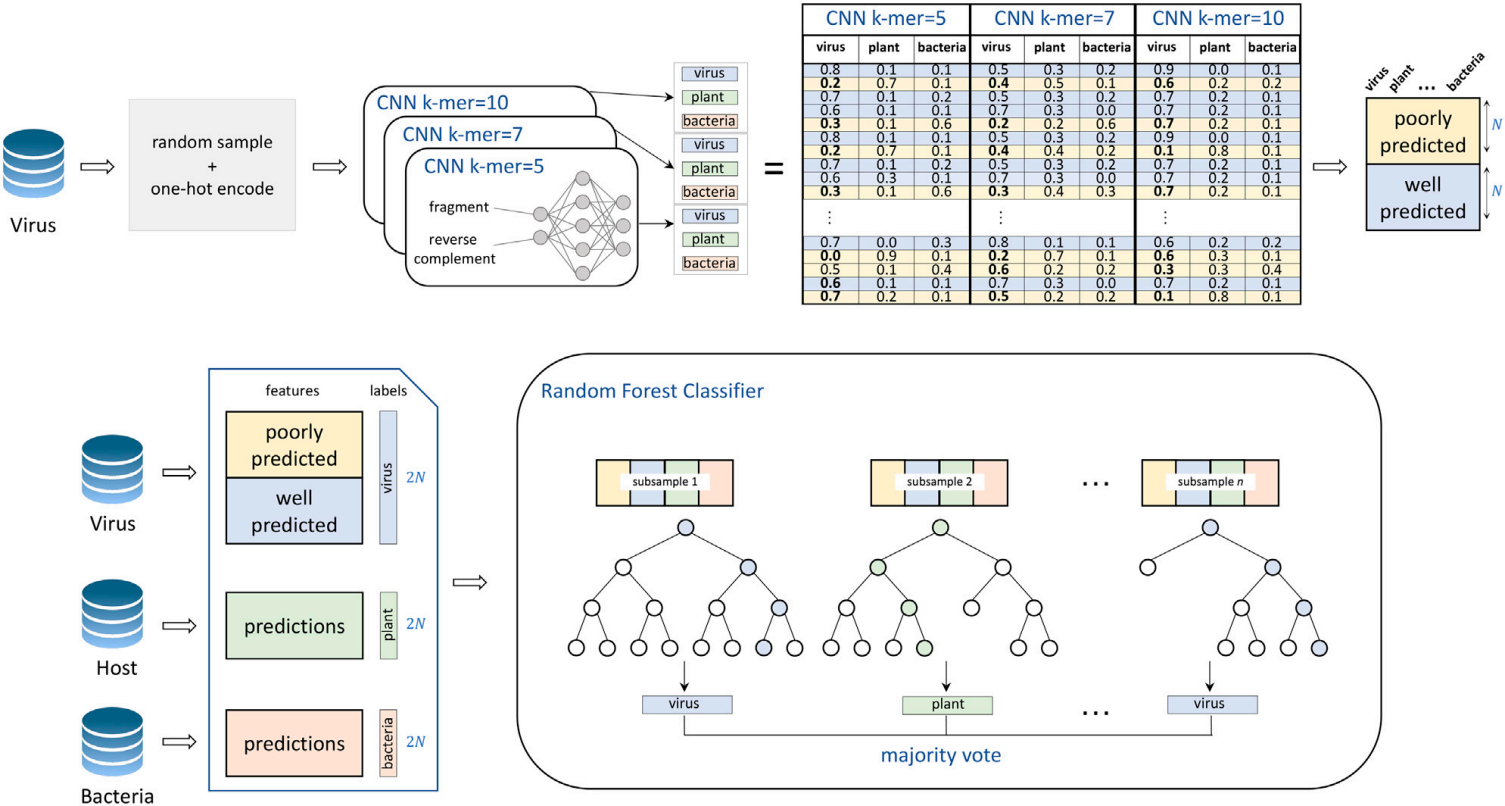
Training of the VirHunter’s machine learning module. The individual network predictions are subsetted to contain an equal number of both poorly predicted (prediction value for viral class < 0.8 ) and well-predicted (prediction value ≥ 0.8 ) viral fragments (with the goal to overselect poor predictions relative to their overall frequency in order to drive the model to recognize even completely novel viruses). The random forest classifier uses these subsetted predictions for its training.

VirHunter architecture was defined with two main components the first component is a multi-path neural network shown in Figure 1, and the second component is a machine learning classification module shown in Figure 2.

*1. Neural network component.* The neural network module follows a k-mer-based approach. To alleviate a potential difficulty related to the choice of k , VirHunter implements a multi-model solution for k=5, 7 , and 10 (see Figure 1), with three independent CNN models having the same architecture. These values of k were chosen based on the accuracy of the individual CNN networks in the family leave-out experiment (see Supplemental Figure S1). The genomic DNA sequence and its reverse complement for each n-size fragment are transformed from nucleotides (in ACGTN alphabet) to an n × 4 one-hot encoded array as presented in *Data Preprocessing*. A convolution layer with leaky rectified linear unit activation function ( a=0.1 ) and global max-pool and dropout layers are then applied independently to the forward fragments and their paired reverse-complement versions. The use of dropout layers was introduced to alleviate the issue of overfitting. Models with k=5,7 have the convolution layer with 256 filters, while the model for k=10 has 512 filters. The two resulting vectors for the forward- and reverse-complement fragments are then concatenated. Finally, two dense layers are applied. The first dense layer has the number of units equal to 256 for the paths with k=5,7 and 512 for the path with k=10 . It employs a rectified linear unit activation function. The second dense layer has three units and uses the softmax activation function to enable three-class classification.

*2. Random Forest component.* The second module of the VirHunter implements a random forest classifier (see Figure 2) with the goal to aggregate the predictions from three neural networks. The classifier receives nine real-valued predictions from the multi-network module (three per network) and outputs one of the three classes using the majority vote implementation of random forest. The random forest classifier was chosen over other approaches such as linear regression and simple voting, based on performance (data not shown).

### Training

The neural network and machine learning modules were trained separately for each of the three plant host species (peach, grapevine, and sugar beet) and for fragment sizes n of 500 and 1,000.

The training dataset for the CNN module was built as presented in *Data Preprocessing*. Training batches with size 512 were prepared in a balanced manner across the three classes (virus, plant, and bacteria) from the training dataset and are split into training and validation with the ratio of 9:1. Each of the three individual networks was trained for 10 epochs, followed by 1 epoch of training on the validation set to take into account all the data.

For training and testing the machine learning components, predictions for the three trained networks were obtained on 100,000 randomly selected fragments of size n from each V and B. Likewise, 100,000 fragments of size n were randomly sampled from H , following the ratio described in *Data Preprocessing*. Predictions for random viral fragments were further subsetted in the following manner. We split the test dataset viral fragments into those having good quality predictions (prediction value for viral class ≥ 0.8 ) and low-quality predictions (prediction value < 0.8 ) and maintained 10,000 randomly selected fragments from each category, yielding 20,000 predictions. These 20,000 predictions were further selected for plant host H and bacterial B fragments. The resulting dataset with three predictions for each of 60,000 fragments was further split in train and test datasets with 2:1 ratio, and the machine learning module was trained with parameters max_depth = 5, n_estimators = 10, max_features = 1, and max_samples = 0.2.

We verified that overfitting was successfully circumvented by the individual CNN networks that compose the neural network component of our model by comparing the accuracy on validation and test datasets obtained by these individual networks trained on families in the leave-out experiment for peach (see Supplementary Table S9). No significant difference was observed.

### Contig Classification

VirHunter trained on fragments with n =500 was used to classify contigs with length 750<l <1500 , while VirHunter trained on fragments with n =1000  was used to classify contigs with 1500<l  . Indeed, an ORF of 500 nucleotides corresponds to an 18 kDa protein, this size covering the vast majority of viral polymerases, movement proteins, and capsid proteins for plant viruses. Contigs with l <750 were considered as very small for prediction by the smaller of the two models and were discarded.

Each fragment of an input contig was preprocessed following the procedure presented in *Data Preprocessing*. Predictions were produced by the neural network module for each of these one-hot encoded fragments, yielding three probabilities of belonging to a specific class ( V, H, B ). These class probabilities were further processed by the random forest component, resulting in a unique class label for each of the fragments. Finally, given class labels for each of the fragments of the input contig, a vote was applied to decide to which class belongs the whole contig, viral if the number of viral ( V ) fragments is greater than those from H and from B , host if the number of host ( H ) fragments is greater than those from V and from B , and bacterial otherwise.

## Results

### VirHunter Outperforms State-of-the-Art Tools on Family Leave-Out Datasets

VirHunter was trained on GPU (Nvidia Tesla T4) with n =1000  for 31 family leave-out datasets and three different plant datasets (peach, grapevine, and sugar beet), resulting in 63 leave-out models. The test datasets were prepared by random sampling of 30,000 fragments with n =1000  from the corresponding left-aside families of viral sequences, bacteria, and plant.

**FIGURE 3:**
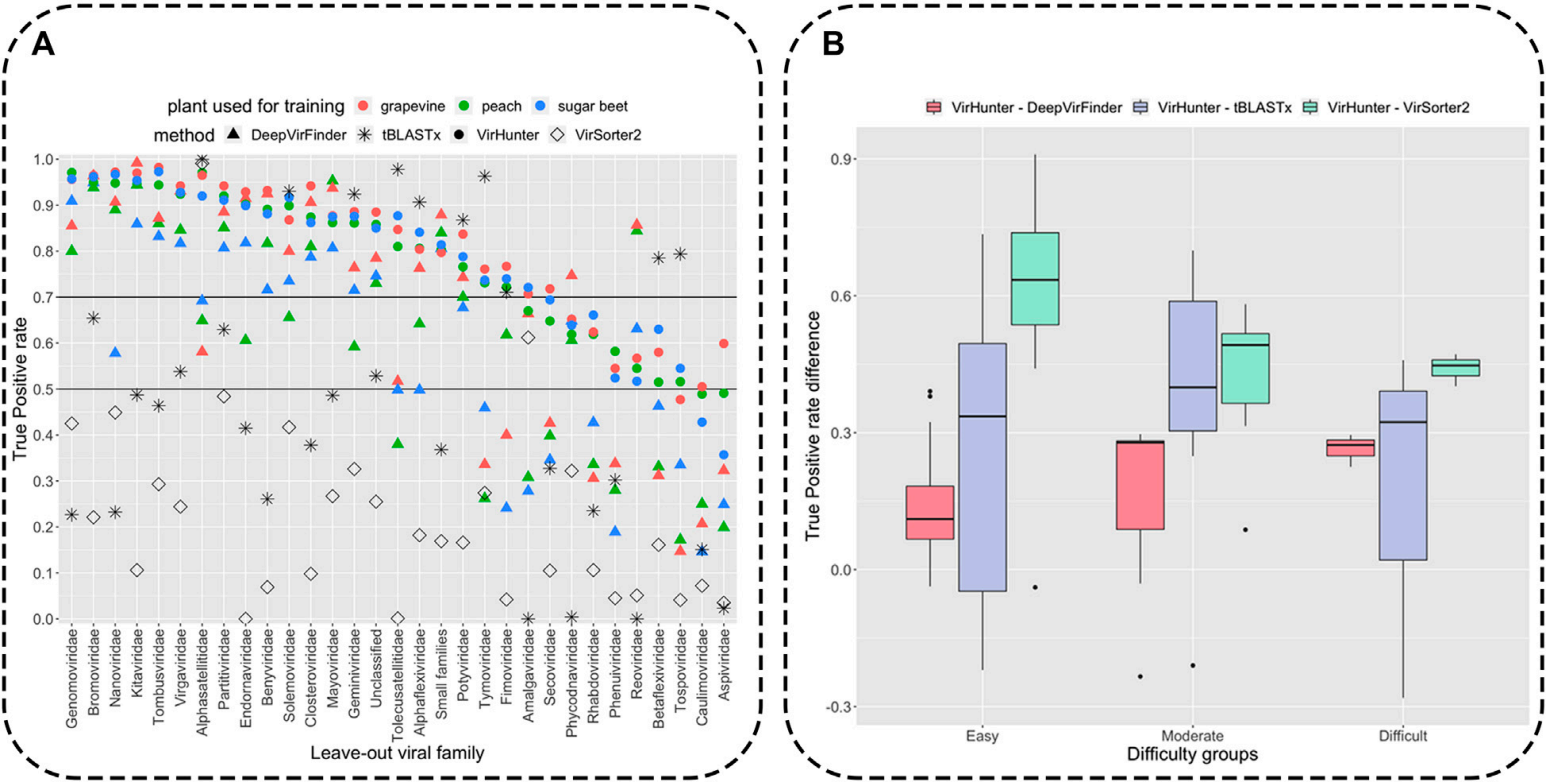
Detection of novel viral fragments in the family leave-out setup. Panel **(A)**: Results for the percent of correctly classified fragments (out of 10,000 ) with length n =1000  from the corresponding left-aside families. VirHunter results are depicted by circles, tBLASTx by stars, DeepVirFinder by triangles, and VirSorter2 by diamonds. Black lines represent thresholds separating families into three difficulty groups for VirHunter as follows: easy to classify (minimum TP rate across the three plants >0.7 ), difficult to classify (minimum TP rate <0.5 ), and moderately difficult to classify (minimum TP rate between 0.5 and 0.7 ). Panel **(B)**: Differences in the True Positive rate between VirHunter, DeepVirFinder (red), tBLASTx (blue), and VirSorter2 (green).

Classification results for the viral fragments by VirHunter in this family leave-out experiment are shown in Figure 3 and in Supplementary Tables S2, S3. We compared the capacity of VirHunter to detect novel viruses in the family leave-out setting with the BLAST-based approach on one hand and two state-of-the-art machine learning methods, DeepVirFinder and VirSorter2, on the other hand as also shown in Figure 3. Briefly, each test dataset was aligned using tBLASTx (v2.12.0), preserving one best hit with parameters -max_target_seqs 1 -max_hsps 1, against the respective virus database with the leave-out family removed, and filtered by e-value $<10^{-10}$ , percent identity >0.5 , and alignment length >50 amino acids (see results in Supplementary Table S4) in order to emulate the annotation workflow without manual inspection; DeepVirFinder was trained on the same 31 leave-out datasets but excluding bacterial fragments from the training dataset since this method provides the possibility to have only two class labels and using the recommended parameters (Ren et al., 2020) on 10 CPUs Intel Xeon CPU E5-2630 v4 (see results in Supplementary Table S5); VirSorter2 was evaluated on each test dataset using pretrained models provided by authors (see results in Supplementary Table S6).

Variability of correct classification was observed for viral fragments of different left-out families for all three methods as shown in Figure 3 (see for detailed results in Supplementary Tables S2–S4). We have split the families into three groups according to the lowest True Positive (TP) rate of VirHunter across the three plant host species: 21 “easy to classify” (TP rate >0.7 ), 7 “moderately difficult to classify” (TP rate between 0.5 and 0.7 ), and 3 “difficult to classify” (TP rate <0.5 ). VirHunter almost systematically outperformed DeepVirFinder in terms of TPs (virus fragments from the leave-out family classified as being viral). In total, there are four exceptions, namely, *Reoviridae*, *Mayoviridae*, *Phycodnaviridae*, and *small families*, out of which *Reoviridae* presented a considerable performance gap. After inspection, it appeared that VirHunter’s false negatives for these four families mostly corresponded to viral fragments being classified as bacteria. This is possibly due to the fact that *Mayoviridae* are bacteriophages, *Reoviridae* concern a very wide range of hosts and present characteristics of bacteriophages [likely evolutionary relationship to the *Cystovirus* family of bacteriophage (Guglielmi et al., 2006)], while the *small families* contain a wide variety of viruses, and bacteriophages are one among them (*Mitoviridae*). This is to be counterbalanced by the fact that being trained only on plant and virus sequences due to the 2-class approach, DeepVirFinder systematically erroneously considers the majority of bacterial fragments as being viral (see Supplementary Table S4). As for the *Phycodnaviridae* family, it contains dsDNA viruses, which could potentially have contributed to the poorer performance of VirHunter relatively to DeepVirFinder for two of the host species. Altogether, VirHunter has shown consistently better capacity to detect novel viruses than DeepVirFinder.

Of note is also the difference in time requirement for training the VirHunter and DeepVirFinder models. On average, training a full model for one leave-out family for one plant host required 11 h for VirHunter (three CNNs, each for both fragment sizes 500 and 1000 – 6 CNNs in total—and the random forest) and 72 h for DeepVirFinder (four CNNs for fragment sizes 150, 300, 500 , and 1000 ).

Compared to both VirHunter and DeepVirFinder, VirSorter2 has shown poorer performance in the family leave-out setup on all the families except two. Indeed, the TP rate was below 0.5 threshold for all families except for the *Amalgaviridiae* and the *Alphasatellitidae*. For the former, VirSorter outperformed DeepVirFinder, while showing poorer results than VirHunter, while for latter it was the best performing method together with tBLASTx (see Panel A of Figure 3).

As shown in Figure 3, despite the reasonably permissive filtering criteria, tBLASTx shows best results comparable with VirHunter and for certain families exhibits particularly poor performance relative to the two machine learning methods. For the “easy to classify” families, the difference was mostly in favor of VirHunter, sometimes drastically (see for example, *Nanoviridae* and *Genomoviridae* in Panel A and the boxplot in Panel B). In seven cases, tBLASTx outperformed VirHunter, but this difference was mostly marginal (5.8% difference in TP rate on average), the outlier being *Tolecusatellitidae* and *Tymoviridae*, where the gain in favor of tBLASTx was the strongest. For the “moderately difficult to classify” families, VirHunter had a higher TP rate than tBLASTx in all cases. For the three “difficult to classify” families, even if VirHunter’s performance was globally low, it still outperformed tBLASTx, with the notable exception of *Tospoviridae*. Altogether, VirHunter has shown consistently better results than that of tBLASTx, for which the TP rate was frequently below the threshold 0.5 (16 families out of 31).

As for the capacity to correctly classify bacterial fragments, VirHunter has shown a systematically high TP rate, ranging from 0.958 to 0.983, across all the leave-out experiments. As for plant fragments, the TP rate was also satisfactory, sugar beet TP from 0.950 to 0.961, grapevine TP from 0.983 to 0.991, and peach TP from 0.983 to 0.989 (see columns “Bacteria” and “Plant” in Supplementary Table S2).

### Plant Fragments Are Accurately Classified When VirHunter Is Trained on Phylogenetically Close Plant Species

VirHunter was trained independently with n=1000 for three selected plants (peach, grapevine, and sugar beet) and all the downloaded viruses and bacteria, generated as described in *Data Preprocessing*, yielding three models.

We cross-evaluated VirHunter’s ability to correctly predict fragments from the plant absent in the training by sampling from the three studied plants, and 10,000 random fragments with n =1000  were selected. Those three plant test datasets were supplemented with two datasets with n =1000  , sampled randomly from all viral sequences and from bacteria, respectively.

As previously described (see *VirHunter Outperforms State-of-the art Tools on Family Leave-out Datasets*), plant fragments, coming from the same plant that the models were trained on, are consistently well classified for all the three models with the TP rate ranging between 0.95 (“sugar beet” model tested on random fragments from the sugar beet genome) and 0.99 (the “peach” model tested on random fragments from the peach genome) as shown in Table 1. When the plant host species used for training the model is reasonably phylogenetically close to the one of the test datasets, the impact on the TP rate is not very important. For example, the “peach” model tested on random fragments from the grapevine genome still produces the TP rate of 0.9, and the “grapevine” model tested on peach fragments gives the TP rate of 0.836. However, both these models generate a lower TP rate when tested on random fragments from the more phylogenetically distant sugar beet fragments, 0.827 and 0.781, for the “peach” and “grapevine” models, respectively. Similarly, the “sugar beet” model performs less well for both peach and grapevine random fragments, with TP rates of 0.854 and 0.887, respectively.

**TABLE 1:** VirHunter results for prediction of fragments from different plants. Classification results for three plant-specific models of 10,000 fragments for length 1000  randomly drawn from three plants’ reference genomes, from all viral sequences and bacteria are shown. In bold are predictions for the expected class.

Plant used for training	Plant used for testing	Predicted label		
		Plant	Virus	Bacteria
Peach	Peach	**0.988**	0.007	0.006
	Grapevine	**0.892**	0.064	0.044
	Sugar beet	**0.804**	0.113	0.083
	Virus	0.002	**0.996**	0.002
	Bacteria	0.005	0.017	**0.978**
Grapevine	Peach	**0.845**	0.106	0.005
	Grapevine	**0.986**	0.011	0.004
	Sugar beet	**0.78**	0.148	0.072
	Virus	0.002	**0.997**	0.002
	Bacteria	0.007	0.021	**0.973**
Sugar beet	Peach	**0.824**	0.132	0.045
	Grapevine	**0.878**	0.087	0.035
	Sugar beet	**0.956**	0.018	0.026
	Virus	0.002	**0.996**	0.002
	Bacteria	0.012	0.019	**0.969**

The three plants used for training models are phylogenetically distant from one another as they belong to different families, sugar beet belongs to the *Amaranthaceae* family, grapevine belongs to the *Vitaceae* family, and peach to the *Rosaceae* family; all the three are *eudicots*. Out of these three plants, sugar beet is the outlier. Peach and grapevine belong to the *Rosids* higher clade, while sugar beet belongs to the *Caryophyllids* higher clade. Given the phylogenetic distance, the lower bound of 0.78 for the true positive rate between these three plants is reasonable.

To evaluate how strongly the performance would be affected if the host of RNAseq dataset was to be from an even further phylogenetically removed plant (belonging to the *monocots*), we trained a model on the rice (*Oryza sativa*) dataset that belongs to *monocots* higher clade. As shown in the Supplementary Table S7, the performance drop was coherent with the increase of the phylogenetic distance (TP rate was 0.766, 0.759, and 0.702 for fragments from peach, grapevine, and sugar beet, respectively); however, the recall remained high for both viral and bacterial fragments. These results highlight that when the host of the RNAseq dataset is phylogenetically highly divergent from any of the plants used to train the available models, a new model for a phylogenetically closer plant has to be trained.

### VirHunter Enables Classification of Long Mutated Viral Fragments

To evaluate the potential quality of VirHunter’s predictions on contigs’ classification, we randomly sampled 10,000 long fragments with n ∈ [1500, 2000, 2500, 3000, 4500, 6000] from the whole virus dataset V . Furthermore, to better emulate contigs resulting from assembly of sequencing reads, we applied a point mutation rate m ∈ [0, 0.05, 0.1, 0.15, 0.2] to these long fragments. Classification of the resulting mutated long fragments was performed using models trained for the three plants as described in *VirHunter Enables Classification of Long-Mutated Viral Fragments* and following the procedure for contig classification described in *Contig Classification*.

We observed that VirHunter generated highly accurate predictions for long viral fragments with 0 mutations and that across different fragment sizes (column “Mutation rate” 0 in Supplementary Table S5). The TP rate slowly decreased with the increase of the mutation rate: for example, the average TP rate across different fragment sizes with the mutation rate 0.2 was 0.885 for the “peach” model, 0.924 for the “grapevine” model, and 0.885 for the “sugar beet” model. Moreover, these results were consistent between the three plant host species used to build the models: the “peach” model’s TP rate was 0.944 in average across different fragment lengths and mutation rates, the “grapevine” models’ average TP rate was 0.960 , and the “sugar beet” model’s average TP rate was 0.936 .

### VirHunter Uncovers Expected Novel and Known Viral Contigs in Virome

The capacity of VirHunter to detect novel viral contigs from real RNAseq-sequencing data was evaluated and compared to that of DeepVirFinder and VirSorter2. The 12 virome RNAseq datasets, sampled from peach, grapevine, and sugar beet (see Supplementary Table S1) were assembled as described in *Assembly of RNAseq Datasets and Annotation of Viral Contigs*. To imitate the novel virus discovery setting, we excluded from the virus dataset those viral species that were annotated as present in the studied plant viromes, and models for each plant species were trained accordingly for VirHunter and DeepVirFinder. For example, to train the “grapevine” model, all viral species present in samples from grapevine (Supplementary Table S1 column “Present viruses”) were deleted from the virus dataset. The same procedure was carried out for training the “peach” and “sugar beet” models. VirSorter2 pretrained models were used following the recommendations in Guo et al. (2021).

The assembled contigs >750 nt were analyzed by VirHunter, DeepVirFinder, and VirSorter2 (see Table 2 and Supplementary Table S8). Importantly, VirHunter assigned a viral label to a lower number of contigs than DeepVirFinder in eight out of 12 datasets (“Viral contigs #” under VirHunter and DeepVirFinder columns). These are the contigs that have to undergo additional manual expert analysis. To better understand their nature, we aligned the contigs identified by VirHunter to the BLAST NCBI nucleotide database limited to “Viruses” taxonomic id as was performed for *Assembly of RNAseq Datasets and Annotation of Viral Contigs* analysis. Contigs getting at least one alignment with percent identity >0.5, length >50 amino acids, and e-value < $10^{-10}$ are reported in the column “tBLASTx hits.”

**TABLE 2:** Performance of VirHunter, DeepVirFinder, and VirSorter2 on 12 RNAseq virome datasets. For each of the 12 datasets shown are the number of contigs that were annotated as being viral by experts and the number of contigs in the initial assembly with length >750 . Columns “VirHunter,” “DeepVirFinder,” and “VirSorter2” show predictions run on these contigs by each method. Columns “# detected” show the total number of contigs detected as being viral by each of the two methods, and columns “detected ∩ annotated” indicates how many of these were previously identified by the curators. Finally, the “tBLASTx e-value < 10^−10^” column indicates how many of “# detected” contigs align against viruses for VirHunter.

Dataset ID and plant origin		# Contig >750	# Contig annotated as viral	VirHunter			DeepVirFinder		VirSorter2	
				# detected	Detected ⋂ annotated	tBLASTx hits (e-val < 10^−10^)	# detected	Detected ⋂ annotated	# detected	Detected ⋂ annotated
P1	Peach	1,009	2	35	2	14	45	2	10	1
P2	Peach	415	2	19	2	10	32	2	8	1
P3	Peach	685	2	23	2	10	49	2	7	1
G1	Grapevine	9,154	10	153	10	47	133	6	52	4
G2	Grapevine	17,024	10	178	10	40	131	9	117	6
G3	Grapevine	18,750	20	208	18	59	137	17	142	11
G4	Grapevine	4,332	15	95	14	32	81	11	24	4
G5	Grapevine	19,395	25	262	23	73	302	23	144	8
G6	Grapevine	2,932	15	70	14	30	86	13	26	12
S1	Sugar beet	6,082	11	236	10	48	335	11	28	6
S2	Sugar beet	8,902	16	277	16	49	419	16	37	7
S3	Sugar beet	6,912	11	203	11	51	307	11	21	4

Moreover, for six datasets (P1, P2, P3, G4, S2, and S3) VirHunter and DeepVirFinder have correctly identified contigs that were previously annotated as viral. For four datasets (G1, G2, G3, and G6), VirHunter was able to discover additional 4, 3, 5, and 1 contigs, respectively. However, for two cases (G5 and S1), DeepVirFinder identified one more annotated contig relative to VirHunter. While VirSorter2 exhibited lower overprediction comparted to VirHunter and DeepVirFinder, its ability to correctly identify viral contigs was low, as it detected at best 60% of the expected viral contigs.

Remember that contigs annotated by experts were all removed from the virus dataset used for the training of VirHunter and DeepVirFinder, V . Consequently, strictly from the computational point of view, detection of these contigs as being viral can thus be considered as detection of novel viruses for those tools. Simple tBLASTx alignment of these expertly annotated contigs against V produced variable percent identity, which was as low as 32.4% for a contig from the G1 grapevine dataset and as high as 99% for a contig from the S1 sugar beet dataset (see Supplementary Table S1). According to the classification of Stobbe and Roossinck, (2014), discovery of these viruses could thus be assimilated in our setup with the discovery of “novel viruses from a known family” and potentially of “completely novel viruses.”

Moreover, it is possible that at least some potentially novel viruses were missed during expert annotation and that the overprediction in columns “# detected” and “tBLASTx hits” (e-val < $10^{-10}$ ) is lower in reality. Indeed, a large number of unidentified novel viruses have been recently shown to be present in public RNAseq datasets by Edgar et al. (2021), where the authors have identified $10^{5}$ novel RNA viruses. Finally, of note is the considerable gain of time left for expert curation of contigs by approaches similar to that presented in *Assembly of RNAseq Datasets and Annotation of Viral Contigs*, given the numbers in the “# detected” column, where VirHunter has shown improvement over DeepVirFinder in eight out of 12 datasets.

## Discussion

High-throughput sequencing (HTS) is capable of broad virus detection for both known and unknown viruses in a variety of hosts and habitats. It has been successfully applied for novel virus discovery in many agricultural crops, leading to the current drive to apply this technology routinely for plant health diagnostics. For this, efficient and precise methods for HTS-based virus detection and discovery are essential.

RNA viruses are the most abundant pathogens infecting plants. However, RNA plant virus detection using HTS presents a number of challenges due to their genetic diversity, lack of conserved regions across viral species, short genome lengths, high mutation rate, and incomplete knowledge present in reference databases. To address this challenge, we developed a novel deep learning method, VirHunter, to detect novel and known plant viruses in RNAseq datasets.

VirHunter is particularly well-suited for the discovery of novel viruses as it was exemplified on 31 synthetic leave-out family datasets, where VirHunter systematically outperformed DeepVirFinder and VirSorter2, reference machine learning tools for virus detection. When compared with the standard tBLASTx approach, we have shown that for most (21 out of 31) leave-out families, VirHunter obtained a higher TP rate. In six cases, tBLASTx was slightly better (5.8% on average). However, there remained four cases where we have seen a much worse performance in VirHunter results. For these specific families, it can be noted that they are particularly well-suited to the alignment-based virus identification, for example, *Alphasatellitidae* viruses carry high sequence similarity to *Geminiviridae* (which was confirmed by the majority of tBLASTx hits).

We have shown that the 3-class classification design of VirHunter, accounting for possible bacterial contamination, was justified by evaluating how such contaminating contigs would be classified. Not surprisingly, VirHunter efficiently dealt with bacterial contamination, while DeepVirFinder classified bacteria mostly (65%) as viruses, which should have been “plants” if the goal is to identify viruses. We have also demonstrated that VirHunter is also perfectly suited for the detection of known divergent viruses, by evaluating classification accuracy on contigs with progressively increasing the mutation rate.

Note the fact that VirHunter is designed to be trained separately for a specific plant host species. However, classification of plant contigs still remained reasonable (minimum 0.78 TP rate) when we performed a cross-evaluation by classifying sequences coming from three phylogenetically distant plants (peach, grapevine, and sugar beet) by each of the three models. As expected, VirHunter performed better, when the plants it was trained and tested on were phylogenetically closer: grapevine and peach belong to the same *rosids* higher clade resulted in better mutual predictions, while sugar beet as an outgroup belonging to the *caryophyllids* higher clade has shown a relative drop in performance. All these three plants are *eudicots* (Pin 2012). When the model was trained on an even further phylogenetically distant plant, rice that belongs to *monocots* and tested on fragments from peach, grapevine, and sugar beet, the classification accuracy of VirHunter was expectedly lower. Together this implies that to classify contigs from an RNAseq experiment, using a pretrained model trained on the exact same plant species as the host of the experimental dataset is not mandatory, but it is preferable to use one trained on a phylogenetically close plant, ideally from the same family and at least belonging to the same *eudicots/monocots* higher clade. A possible avenue to explore in the future work is the feasibility of transfer learning (Eraslan et al., 2019), to enable fast on-demand retraining for a new plant or building a generalistic plant model.

Finally, we validated VirHunter’s capacity to detect novel viruses on 12 newly acquired RNAseq datasets for peach, grapevine, and sugar beet. In these datasets, VirHunter detected at least 90% (73% for DeepVirFinder and 26% for VirSorter2) of all expert-annotated viral contigs, and in seven datasets it was 100%. Another contribution is the low rate of false positives generated by VirHunter, leaving from 19 to 277 contigs depending on the dataset to be inspected by an expert. These results indicate that VirHunter efficiently reduces the number of contigs requiring manual expert curation.

In conclusion, we have shown that VirHunter can be used to streamline the analyses of plant HTS-acquired viromes and is particularly well suited for the detection of novel viral contigs, in RNAseq datasets.

## Data Availability

The datasets presented in this study can be found in online repositories. The names of the repository/repositories and accession number(s) can be found in the article/Supplementary Material.
