# Supplementary material for: VirHunter: A Deep Learning-Based Method for Detection of Novel RNA Viruses in Plant Sequencing Data
Source: Front Bioinform. 2022 May 13;2:867111. doi: 10.3389/fbinf.2022.867111 (PMC9580956; doi:10.3389/fbinf.2022.867111)
Supplement: Supplementary file 1 [file DataSheet1.pdf]

Supplementary Material for "VirHunter: a deep learning-based method for detection of novel RNA viruses in plant sequencing data"

Grigorii Sukhorukov[1,2], Maryam Khalili[3], Olivier Gascuel[4], Thierry Candresse[3], Armelle Marais-Colombel[3], Macha Nikolski[1,2]

1 CNRS, IBGC, UMR 5095, Université de Bordeaux, Bordeaux, France

2 Bordeaux Bioinformatics Center, Université de Bordeaux, Bordeaux, France

3 Université de Bordeaux, INRAE, UMR BFP, CS20032, CEDEX, Villenave d'Ornon, France

4 Institut de Systématique, Biodiversité, Evolution (ISYEB - UMR7205, Muséum National d'Histoire Naturelle, CNRS, SU, EPHE, UA), Paris, France

The choice of k-mer size for individual networks was also done deliberately (see Supplementary Figure 1), as we selected $k = 5$ for the first network as the smallest k having acceptable results, $k = 10$ for having good results especially on families that are difficult to classify and $k = 7$ for complementarity, 7 not being a prime factor of 5 and 10.

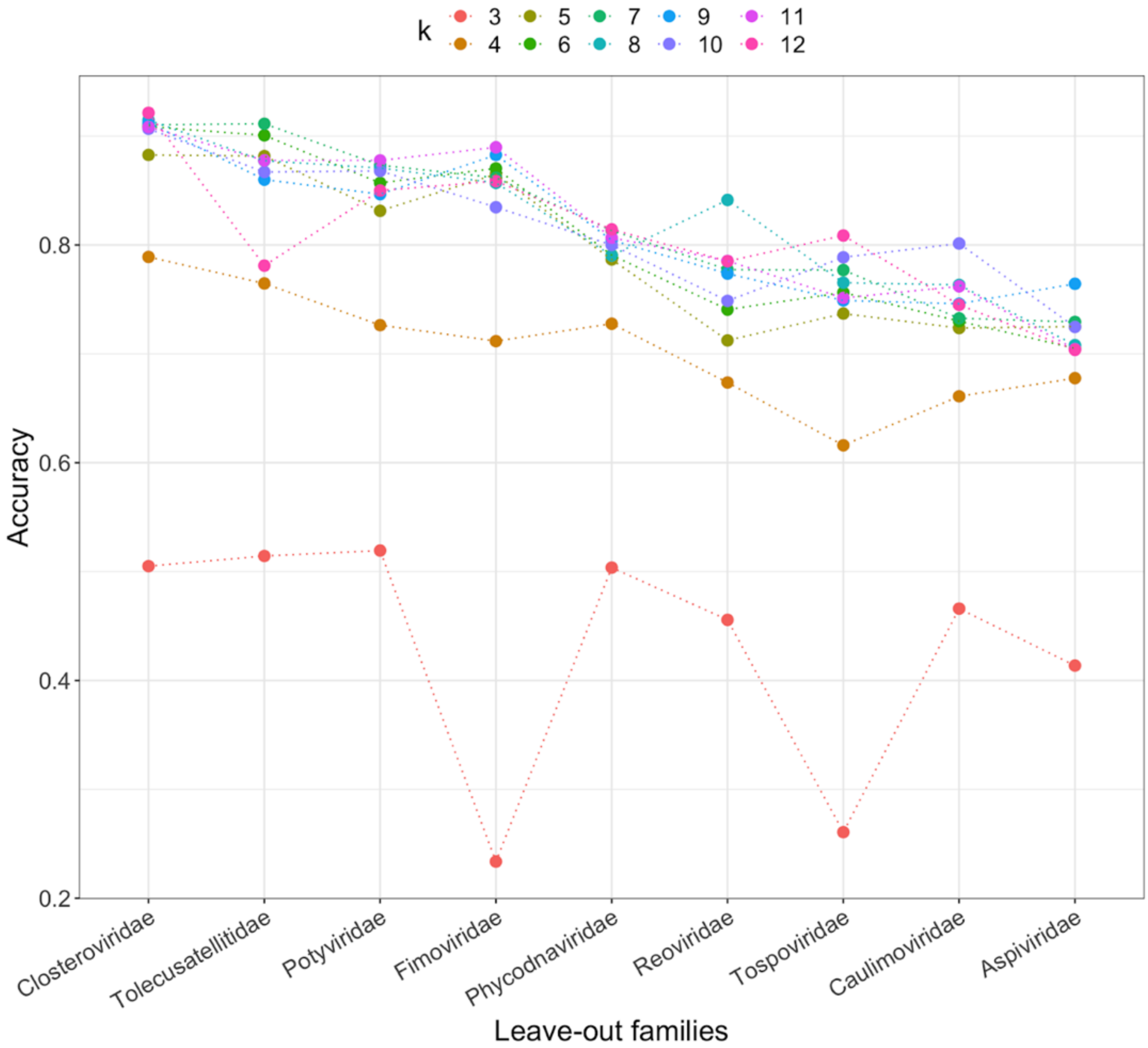

Supplementary Figure 1. Classification accuracy of individual CNN networks in the family leave-out setup for different values of k. Shown is the classification accuracy of 10 individual networks for $k = [3..12]$ calculated for the 3 test sets (10,000 fragments from each class V, B, H) with length $n = 1000$ from the 8 left-aside families. The X axis shows these 8 families sorted according to the classification difficulty of VirHunter (same as in Figure 3), and the Y axis shows classification accuracy. Colors represent different k values.

Supplementary Table 1. Description of the newly acquired RNA-Seq datasets. 12 RNASeq datasets coming from 9 virome studies from 3 plants are presented. Shown are viruses identified in the datasets following the annotation procedure in section 2.3.

DOI study id	short name	File name	Host	Present viruses			
				name	GenBank id	# of contigs > 750 bp in assembly	tBLASTx average identity, %
10.15454/GWDPIN	P1	LIB1_TGCGGCGT-TACCGAGG-AH2C7TDRXY_L001_{R1,R2}.fastq.gz	Prunus persica	Stocky prune virus RNA1	NC_043388	1	66.5
				Stocky prune virus RNA2	NC_043387	1	50.2
	P2	LIB2_CATAATAC-CGTTAGAA-AH2C7TDRXY_L001_{R1,R2}.fastq.gz	Prunus persica	Apricot latent ringspot virus RNA1	NC_043411.1	1	62.5
				Apricot latent ringspot virus RNA2	---	1	78.8
	P3	LIB3_GATCTATC-AGCCTCAT-AH2C7TDRXY_L001_{R1,R2}.fastq.gz	Prunus persica	Myrobalan latent ringspot virus RNA1	---	1	61.7
				Myrobalan latent ringspot virus RNA2	---	1	80.8
10.15454/MK1JIW	S1	305S_{R1,R2}_001.fastq.gz	Beta vulgaris	Beet yellows virus	NC_001598	2	78.3

				Beet chlorosis virus	NC_002766	1	74.1
				Beet mosaic virus	NC_005304	2	99.8
				Beta vulgaris mitovirus 1	BK010442	1	50.4
				Beet cryptic virus 2 RNA1	NC_038846	1	75.0
				Beet cryptic virus 2 RNA2a	NC_038845	1	52.9
				Beet cryptic virus 2 RNA2b	NC_038847	1	49.4
				Beta vulgaris satellite virus 1A	MT227166	1	43.3
				Beta vulgaris satellite virus 1B	MT227167	1	42.2
	S2	333A_{R1,R2}_001.fastq.gz	Beta vulgaris	Beet yellows virus	NC_001598	1	73.9
				Beet mild yellowing virus	NC_003491	4	65.3

				Beet chlorosis virus	NC_002766	3	69.1
				Beet mosaic virus	NC_005304	7	97.3
				Beta vulgaris mitovirus 1	BK010442	1	50.4
	S3	428A_{R1,R2}_001.fastq.gz	Beta vulgaris	Beet yellows virus	NC_001598	2	78.1
				Beet mosaic virus	NC_005304	6	98.7
				Beta vulgaris mitovirus 1	BK010442	1	50.4
				Beet cryptic virus 2 RNA1	NC_038846	1	73.0
				Beet cryptic virus 2 RNA2a	NC_038845	1	52.9
10.15454/KUYAT9	G1	I30-2_CTGAAGCT-AGGCTATA_L007_{R1,R2}.fastq.gz	Vitis Vinifera	Grapevine rupestris stem pitting-associated virus	NC_001948	9	66.8
				Grapevine leafroll-associated virus 2	NC_007448	1	64.0

	G2	I30-2_TGACCA_L007_{R1,R2}.fastq.gz	Vitis Vinifera	Grapevine rupestris stem pitting-associated virus	NC_001948	9	67.8
				Grapevine leafroll-associated virus 2	NC_007448	1	47.9
	G3	I33_CTGAAGCT-TCAGAGCC_L007_{R1,R2}.fastq.gz	Vitis Vinifera	Grapevine rupestris stem pitting-associated virus	NC_001948	18	62.2
				Grapevine Red Globe virus	NC_030693	2	63.5
	G4	I33_CAGATC_L007_{R1,R2}.fastq.gz	Vitis Vinifera	Grapevine rupestris stem pitting-associated virus	NC_001948	12	62.2
				Grapevine Red Globe virus	NC_030693	3	63.5
	G5	TT2017-1_ATGTCA_L007_{R1,R2}.fastq.gz	Vitis Vinifera	Grapevine rupestris stem pitting-	NC_001948	3	74.3

				associated virus			
				Grapevine leafroll-associated virus 1	NC_016509	4	39.0
				Grapevine leafroll-associated virus 3	NC_004667	2	65.6
				Grapevine leafroll-associated virus 7	NC_016436	10	48.8
				Grapevine satellite virus	NC_021480	1	55.7
				Grapevine Kizil Sapak virus	MN172165	5	46.0
	G6	I33_CTGAAGCT-TCAGAGCC_L007_{R1,R2}.fastq.gz	Vitis Vinifera	Grapevine rupestris stem pitting-associated virus	NC_001948	5	68.8
				Grapevine leafroll-associated virus 1	NC_016509	1	53.2
				Grapevine leafroll-	NC_004667	1	92.4

				associated virus 3			
				Grapevine leafroll-associated virus 7	NC_016436	2	58.1
				Grapevine satellite virus	NC_021480	1	55.7
				Grapevine Kizil Sapak virus	MN172165	5	45.4

Supplementary Table 2. VirHunter results for the detection of novel viral fragments in the family leave-out setup. 'True label' corresponds to the origin of the fragments, while 'Predicted label' row corresponds to the label predicted by VirHunter for those fragments.

Plant used for training	True label / Predicted label / Leave-out family	Plant			Virus			Bacteria		
		Plant	Virus	Bacteria	Plant	Virus	Bacteria	Plant	Virus	Bacteria
Peach	Alphaflexiviridae	9,870	90	40	1,870	8,060	80	50	190	9,760
	Alphasatellitidae	9,880	60	60	250	9,710	40	50	210	9,740
	Amalgaviridae	9,890	80	30	2,880	6,700	420	80	180	9,740
	Aspiviridae	9,850	90	50	5,010	4,910	80	30	300	9,670
	Benyviridae	9,900	50	50	540	8,910	540	80	220	9,700
	Betaflexiviridae	9,870	60	70	4,630	5,150	230	40	190	9,770
	Bromoviridae	9,900	60	40	50	9,500	450	50	150	9,790
	Caulimoviridae	9,860	80	60	3,890	4,890	1,210	50	220	9,730
	Closteroviridae	9,850	80	60	520	8,740	740	80	190	9,720
	Endornaviridae	9,850	80	80	840	9,020	140	50	210	9,740
	Fimoviridae	9,870	90	40	1,680	7,220	1,110	60	240	9,690
	Geminiviridae	9,830	140	30	1,270	8,610	120	70	230	9,700
	Genomoviridae	9,880	80	40	140	9,710	140	60	250	9,690
	Kitaviridae	9,900	50	50	50	9,460	480	60	130	9,810
	Mayoviridae	9,890	70	40	80	8,620	1,310	80	300	9,610
	Nanoviridae	9,850	90	70	210	9,480	300	40	200	9,750
	Partitiviridae	9,830	110	60	400	9,200	400	60	230	9,710
	Phenuiviridae	9,860	70	70	4,140	5,820	40	60	220	9,720
	Phycodnaviridae	9,840	100	60	1,330	6,190	2,480	70	160	9,770
	Potyviridae	9,860	90	50	2,050	7,660	280	140	230	9,630
	Reoviridae	9,880	70	50	220	5,450	4,330	50	210	9,730
	Rhabdoviridae	9,870	70	60	3,670	6,190	130	50	150	9,790
	Secoviridae	9,900	70	30	3,350	6,480	180	90	250	9,660
	Small families	9,880	50	70	210	8,040	1,740	60	160	9,780
	Solemoviridae	9,820	120	60	620	8,990	390	60	180	9,770
	Tolecusatellitidae	9,840	80	80	1,740	8,100	160	50	200	9,750
	Tombusviridae	9,890	70	40	290	9,440	270	80	160	9,770
	Tospoviridae	9,900	50	50	4,610	5,160	230	60	210	9,730
	Tymoviridae	9,860	90	40	1,260	7,310	1,420	70	210	9,720
	Unclassified	9,860	90	50	630	8,580	800	50	220	9,730
	Virgaviridae	9,850	90	60	380	9,240	380	100	240	9,660
Grapevine	Alphaflexiviridae	9,870	110	30	1,860	8,040	100	40	200	9,750
	Alphasatellitidae	9,890	90	20	300	9,650	40	40	160	9,800

	Amalgaviridae	9,850	120	30	2,770	7,070	160	60	230	9,710
	Aspiviridae	9,850	130	30	3,910	5,990	100	40	350	9,610
	Benyviridae	9,870	100	30	20	9,320	660	70	170	9,750
	Betaflexiviridae	9,860	100	30	3,960	5,800	240	40	250	9,710
	Bromoviridae	9,860	110	30	40	9,650	300	100	190	9,700
	Caulimoviridae	9,890	80	30	3,820	5,050	1,130	30	240	9,720
	Closteroviridae	9,830	150	30	220	9,420	350	80	240	9,680
	Endornaviridae	9,870	100	30	220	9,290	490	50	160	9,800
	Fimoviridae	9,840	140	20	1,530	7,670	800	70	150	9,780
	Geminiviridae	9,860	100	30	1,000	8,860	140	50	200	9,750
	Genomoviridae	9,890	80	30	140	9,560	310	60	180	9,760
	Kitaviridae	9,860	110	30	0	9,700	290	70	170	9,760
	Mayoviridae	9,850	120	30	30	8,770	1,200	80	240	9,680
	Nanoviridae	9,830	140	30	20	9,720	260	60	230	9,710
	Partitiviridae	9,850	110	30	320	9,420	260	50	230	9,720
	Phenuiviridae	9,860	100	40	4,450	5,450	100	50	180	9,770
	Phycodnaviridae	9,870	90	40	710	6,520	2,760	70	190	9,740
	Potyviridae	9,890	80	30	1,300	8,370	330	50	180	9,760
	Reoviridae	9,870	110	30	30	5,670	4,300	70	250	9,680
	Rhabdoviridae	9,890	90	20	3,700	6,240	60	70	160	9,770
	Secoviridae	9,830	130	40	2,650	7,180	170	60	240	9,700
	Small families	9,860	100	30	290	7,970	1,740	30	160	9,810
	Solemoviridae	9,850	120	30	990	8,680	330	50	180	9,770
	Tolecusatellitidae	9,860	100	40	1,410	8,470	110	40	240	9,720
	Tombusviridae	9,840	130	30	110	9,820	70	50	210	9,750
	Tospoviridae	9,840	130	20	5,100	4,770	130	70	180	9,760
	Tymoviridae	9,830	140	30	1,050	7,610	1,340	50	270	9,680
	Unclassified	9,830	130	40	460	8,850	690	70	290	9,640
	Virgaviridae	9,850	120	30	170	9,420	400	50	310	9,630
Sugar beet	Alphaflexiviridae	9,550	180	260	1,480	8,410	100	100	200	9,700
	Alphasatellitidae	9,540	210	240	760	9,200	50	130	210	9,650
	Amalgaviridae	9,510	210	280	2,420	7,210	370	70	180	9,750
	Aspiviridae	9,560	180	260	6,360	3,570	70	120	150	9,730
	Benyviridae	9,580	170	250	630	8,810	560	90	200	9,710
	Betaflexiviridae	9,540	210	250	3,550	6,300	150	80	330	9,590
	Bromoviridae	9,570	180	250	170	9,620	210	130	150	9,730
	Caulimoviridae	9,530	200	270	4,860	4,280	860	90	230	9,680
	Closteroviridae	9,560	160	280	790	8,620	590	90	220	9,690
	Endornaviridae	9,540	180	290	580	8,990	420	90	190	9,730
	Fimoviridae	9,540	180	280	1,480	7,400	1,110	90	220	9,690
	Geminiviridae	9,530	220	240	1,200	8,760	50	160	260	9,580

	Genomoviridae	9,580	160	260	150	9,570	280	80	170	9,750
	Kitaviridae	9,520	220	260	180	9,540	280	110	220	9,670
	Mayoviridae	9,520	210	270	10	8,750	1,230	80	270	9,650
	Nanoviridae	9,600	130	260	190	9,670	140	130	130	9,730
	Partitiviridae	9,500	230	270	550	9,110	340	130	220	9,660
	Phenuiviridae	9,540	200	260	4,680	5,240	80	80	280	9,650
	Phycodnaviridae	9,560	160	280	1,310	6,390	2,300	90	160	9,750
	Potyviridae	9,550	180	270	1,760	7,880	350	100	170	9,730
	Reoviridae	9,610	120	270	550	5,170	4,280	100	150	9,750
	Rhabdoviridae	9,530	200	270	3,340	6,610	50	90	210	9,700
	Secoviridae	9,500	220	280	2,840	6,940	220	100	270	9,630
	Small families	9,550	180	270	460	8,140	1,390	80	200	9,720
	Solemoviridae	9,530	210	260	450	9,170	380	100	210	9,690
	Tolecusatellitidae	9,510	210	280	1,180	8,770	50	120	210	9,660
	Tombusviridae	9,590	170	240	120	9,730	140	90	170	9,730
	Tospoviridae	9,520	220	260	4,110	5,450	440	100	220	9,680
	Tymoviridae	9,530	210	260	520	7,370	2,110	100	260	9,640
	Unclassified	9,580	170	250	760	8,500	730	120	180	9,690
	Virgaviridae	9,560	190	250	380	9,280	340	110	260	9,640

pplementary Table 3. Statistics for VirHunter results for the detection of novel viral fragments in the family leave-out setup.
l the statistics are calculated separately for each of the three classes.

Plant used for train	Statistics for 3 classes / Leave-out family	Plant								Virus								Bacteria							
		TP	FP	TN	FN	TPR	Accuracy	Precision	Recall	TP	FP	TN	FN	TPR	Accuracy	Precision	Recall	TP	FP	TN	FN	TPR	Accuracy	Precision	Recall
Peach	Alphaflexiviridae	9,870	1,920	18,090	130	0.99	0.93	0.84	0.99	8,060	280	19,720	1,950	0.81	0.93	0.97	0.81	9,760	120	19,890	240	0.98	0.99	0.99	0.98
	Alphasatellitidae	9,880	300	19,700	120	0.99	0.99	0.97	0.99	9,710	270	19,730	290	0.97	0.98	0.97	0.97	9,740	100	19,900	260	0.97	0.99	0.99	0.97
	Amalgaviridae	9,890	2,960	17,040	110	0.99	0.90	0.77	0.99	6,700	260	19,740	3,300	0.67	0.88	0.96	0.67	9,740	450	19,550	260	0.97	0.98	0.96	0.97
	Aspiviridae	9,850	5,040	14,960	140	0.99	0.83	0.66	0.99	4,910	390	19,600	5,090	0.49	0.82	0.93	0.49	9,670	130	19,860	330	0.97	0.98	0.99	0.97
	Benyviridae	9,900	620	19,370	100	0.99	0.98	0.94	0.99	8,910	270	19,730	1,080	0.89	0.95	0.97	0.89	9,700	590	19,400	300	0.97	0.97	0.94	0.97
	Betaflexiviridae	9,870	4,670	15,340	130	0.99	0.84	0.68	0.99	5,150	250	19,750	4,860	0.51	0.83	0.95	0.51	9,770	300	19,710	230	0.98	0.98	0.97	0.98
	Bromoviridae	9,900	100	19,890	100	0.99	0.99	0.99	0.99	9,500	210	19,780	500	0.95	0.98	0.98	0.95	9,790	490	19,510	200	0.98	0.98	0.95	0.98
	Caulimoviridae	9,860	3,940	16,050	140	0.99	0.86	0.71	0.99	4,890	300	19,700	5,100	0.49	0.82	0.94	0.49	9,730	1,270	18,720	270	0.97	0.95	0.88	0.97
	Closteroviridae	9,850	600	19,390	140	0.99	0.98	0.94	0.99	8,740	270	19,710	1,260	0.87	0.95	0.97	0.87	9,720	800	19,190	270	0.97	0.96	0.92	0.97
	Endornaviridae	9,850	890	19,110	160	0.98	0.97	0.92	0.98	9,020	290	19,720	980	0.90	0.96	0.97	0.90	9,740	220	19,790	260	0.97	0.98	0.98	0.97
	Fimoviridae	9,870	1,740	18,260	130	0.99	0.94	0.85	0.99	7,220	330	19,660	2,790	0.72	0.90	0.96	0.72	9,690	1,150	18,860	300	0.97	0.95	0.89	0.97
	Geminiviridae	9,830	1,340	18,660	170	0.98	0.95	0.88	0.98	8,610	370	19,630	1,390	0.86	0.94	0.96	0.86	9,700	150	19,850	300	0.97	0.99	0.98	0.97
	Genomoviridae	9,880	200	19,790	120	0.99	0.99	0.98	0.99	9,710	330	19,670	280	0.97	0.98	0.97	0.97	9,690	180	19,810	310	0.97	0.98	0.98	0.97
	Kitaviridae	9,900	110	19,880	100	0.99	0.99	0.99	0.99	9,460	180	19,820	530	0.95	0.98	0.98	0.95	9,810	530	19,460	190	0.98	0.98	0.95	0.98
	Mayoviridae	9,890	160	19,840	110	0.99	0.99	0.98	0.99	8,620	370	19,620	1,390	0.86	0.94	0.96	0.86	9,610	1,350	18,660	380	0.96	0.94	0.88	0.96
	Nanoviridae	9,850	250	19,730	160	0.98	0.99	0.98	0.98	9,480	290	19,710	510	0.95	0.97	0.97	0.95	9,750	370	19,630	240	0.98	0.98	0.96	0.98
	Partitiviridae	9,830	460	19,540	170	0.98	0.98	0.96	0.98	9,200	340	19,660	800	0.92	0.96	0.96	0.92	9,710	460	19,540	290	0.97	0.98	0.95	0.97
	Phenuiviridae	9,860	4,200	15,800	140	0.99	0.86	0.70	0.99	5,820	290	19,710	4,180	0.58	0.85	0.95	0.58	9,720	110	19,890	280	0.97	0.99	0.99	0.97
	Phycodnaviridae	9,840	1,400	18,600	160	0.98	0.95	0.88	0.98	6,190	260	19,740	3,810	0.62	0.86	0.96	0.62	9,770	2,540	17,460	230	0.98	0.91	0.79	0.98
	Potyviridae	9,860	2,190	17,800	140	0.99	0.92	0.82	0.99	7,660	320	19,680	2,330	0.77	0.91	0.96	0.77	9,630	330	19,660	370	0.96	0.98	0.97	0.96
	Reoviridae	9,880	270	19,720	120	0.99	0.99	0.97	0.99	5,450	280	19,710	4,550	0.55	0.84	0.95	0.55	9,730	4,380	15,620	260	0.97	0.85	0.69	0.97
	Rhabdoviridae	9,870	3,720	16,260	130	0.99	0.87	0.73	0.99	6,190	220	19,770	3,800	0.62	0.87	0.97	0.62	9,790	190	19,800	200	0.98	0.99	0.98	0.98
	Secoviridae	9,900	3,440	16,570	100	0.99	0.88	0.74	0.99	6,480	320	19,680	3,530	0.65	0.87	0.95	0.65	9,660	210	19,800	340	0.97	0.98	0.98	0.97
	Small families	9,880	270	19,720	120	0.99	0.99	0.97	0.99	8,040	210	19,790	1,950	0.80	0.93	0.97	0.80	9,780	1,810	18,180	220	0.98	0.93	0.84	0.98
	Solemoviridae	9,820	680	19,330	180	0.98	0.97	0.94	0.98	8,990	300	19,710	1,010	0.90	0.96	0.97	0.90	9,770	450	19,550	240	0.98	0.98	0.96	0.98
	Tolecusatellitidae	9,840	1,790	18,210	160	0.98	0.94	0.85	0.98	8,100	280	19,720	1,900	0.81	0.93	0.97	0.81	9,750	240	19,760	250	0.98	0.98	0.98	0.98
	Tombusviridae	9,890	370	19,640	110	0.99	0.98	0.96	0.99	9,440	230	19,780	560	0.94	0.97	0.98	0.94	9,770	310	19,690	240	0.98	0.98	0.97	0.98
	Tospoviridae	9,900	4,670	15,330	100	0.99	0.84	0.68	0.99	5,160	260	19,740	4,840	0.52	0.83	0.95	0.52	9,730	280	19,720	270	0.97	0.98	0.97	0.97
	Tymoviridae	9,860	1,330	18,660	130	0.99	0.95	0.88	0.99	7,310	300	19,690	2,680	0.73	0.90	0.96	0.73	9,720	1,460	18,520	280	0.97	0.94	0.87	0.97
	Unclassified	9,860	680	19,330	140	0.99	0.97	0.94	0.99	8,580	310	19,690	1,430	0.86	0.94	0.97	0.86	9,730	850	19,160	270	0.97	0.96	0.92	0.97
	Virgaviridae	9,850	480	19,520	150	0.99	0.98	0.95	0.99	9,240	330	19,670	760	0.92	0.96	0.97	0.92	9,660	440	19,560	340	0.97	0.97	0.96	0.97

Grapevine	Alphaflexiviridae	9,870	1,900	18,090	140	0.99	0.93	0.84	0.99	8,040	310	19,690	1,960	0.80	0.92	0.96	0.80	9,750	130	19,880	240	0.98	0.99	0.99	0.98
	Alphasatellitidae	9,890	340	19,650	110	0.99	0.98	0.97	0.99	9,650	250	19,750	340	0.97	0.98	0.97	0.97	9,800	60	19,930	200	0.98	0.99	0.99	0.98
	Amalgaviridae	9,850	2,830	17,170	150	0.99	0.90	0.78	0.99	7,070	350	19,650	2,930	0.71	0.89	0.95	0.71	9,710	190	19,810	290	0.97	0.98	0.98	0.97
	Aspiviridae	9,850	3,950	16,050	160	0.98	0.86	0.71	0.98	5,990	480	19,530	4,010	0.60	0.85	0.93	0.60	9,610	130	19,880	390	0.96	0.98	0.99	0.96
	Benyviridae	9,870	90	19,900	130	0.99	0.99	0.99	0.99	9,320	270	19,720	680	0.93	0.97	0.97	0.93	9,750	690	19,310	240	0.98	0.97	0.93	0.98
	Betaflexiviridae	9,860	4,000	16,000	130	0.99	0.86	0.71	0.99	5,800	350	19,640	4,200	0.58	0.85	0.94	0.58	9,710	270	19,720	290	0.97	0.98	0.97	0.97
	Bromoviridae	9,860	140	19,840	140	0.99	0.99	0.99	0.99	9,650	300	19,690	340	0.97	0.98	0.97	0.97	9,700	330	19,660	290	0.97	0.98	0.97	0.97
	Caulimoviridae	9,890	3,850	16,140	110	0.99	0.87	0.72	0.99	5,050	320	19,670	4,950	0.51	0.82	0.94	0.51	9,720	1,160	18,840	270	0.97	0.95	0.89	0.97
	Closteroviridae	9,830	300	19,690	180	0.98	0.98	0.97	0.98	9,420	390	19,620	570	0.94	0.97	0.96	0.94	9,680	380	19,620	320	0.97	0.98	0.96	0.97
	Endornaviridae	9,870	270	19,740	130	0.99	0.99	0.97	0.99	9,290	260	19,750	710	0.93	0.97	0.97	0.93	9,800	520	19,480	210	0.98	0.98	0.95	0.98
	Fimoviridae	9,840	1,600	18,400	160	0.98	0.94	0.86	0.98	7,670	290	19,710	2,330	0.77	0.91	0.96	0.77	9,780	820	19,180	220	0.98	0.97	0.92	0.98
	Geminiviridae	9,860	1,050	18,950	130	0.99	0.96	0.90	0.99	8,860	300	19,690	1,140	0.89	0.95	0.97	0.89	9,750	170	19,820	250	0.98	0.99	0.98	0.98
	Genomoviridae	9,890	200	19,810	110	0.99	0.99	0.98	0.99	9,560	260	19,740	450	0.96	0.98	0.97	0.96	9,760	340	19,670	240	0.98	0.97	0.97	0.98
	Kitaviridae	9,860	70	19,920	140	0.99	0.99	0.99	0.99	9,700	280	19,720	290	0.97	0.98	0.97	0.97	9,760	320	19,670	240	0.98	0.98	0.97	0.98
	Mayoviridae	9,850	110	19,890	150	0.99	0.99	0.99	0.99	8,770	360	19,640	1,230	0.88	0.95	0.96	0.88	9,680	1,230	18,770	320	0.97	0.95	0.89	0.97
	Nanoviridae	9,830	80	19,920	170	0.98	0.99	0.99	0.98	9,720	370	19,630	280	0.97	0.98	0.96	0.97	9,710	290	19,710	290	0.97	0.98	0.97	0.97
	Partitiviridae	9,850	370	19,630	140	0.99	0.98	0.96	0.99	9,420	340	19,650	580	0.94	0.97	0.97	0.94	9,720	290	19,700	280	0.97	0.98	0.97	0.97
	Phenuiviridae	9,860	4,500	15,500	140	0.99	0.85	0.69	0.99	5,450	280	19,720	4,550	0.55	0.84	0.95	0.55	9,770	140	19,860	230	0.98	0.99	0.99	0.98
	Phycodnaviridae	9,870	780	19,210	130	0.99	0.97	0.93	0.99	6,520	280	19,720	3,470	0.65	0.87	0.96	0.65	9,740	2,800	17,190	260	0.97	0.90	0.78	0.97
	Potyviridae	9,890	1,350	18,640	110	0.99	0.95	0.88	0.99	8,370	260	19,730	1,630	0.84	0.94	0.97	0.84	9,760	360	19,640	230	0.98	0.98	0.96	0.98
	Reoviridae	9,870	100	19,900	140	0.99	0.99	0.99	0.99	5,670	360	19,650	4,330	0.57	0.84	0.94	0.57	9,680	4,330	15,680	320	0.97	0.85	0.69	0.97
	Rhabdoviridae	9,890	3,770	16,230	110	0.99	0.87	0.72	0.99	6,240	250	19,750	3,760	0.62	0.87	0.96	0.62	9,770	80	19,920	230	0.98	0.99	0.99	0.98
	Secoviridae	9,830	2,710	17,290	170	0.98	0.90	0.78	0.98	7,180	370	19,630	2,820	0.72	0.89	0.95	0.72	9,700	210	19,790	300	0.97	0.98	0.98	0.97
	Small families	9,860	320	19,680	130	0.99	0.98	0.97	0.99	7,970	260	19,730	2,030	0.80	0.92	0.97	0.80	9,810	1,770	18,220	190	0.98	0.93	0.85	0.98
	Solemoviridae	9,850	1,040	18,960	150	0.99	0.96	0.90	0.99	8,680	300	19,700	1,320	0.87	0.95	0.97	0.87	9,770	360	19,640	230	0.98	0.98	0.96	0.98
	Tolecusatellitidae	9,860	1,450	18,540	140	0.99	0.95	0.87	0.99	8,470	340	19,660	1,520	0.85	0.94	0.96	0.85	9,720	150	19,840	280	0.97	0.99	0.98	0.97
	Tombusviridae	9,840	160	19,850	160	0.98	0.99	0.98	0.98	9,820	340	19,670	180	0.98	0.98	0.97	0.98	9,750	100	19,900	260	0.97	0.99	0.99	0.97
	Tospoviridae	9,840	5,170	14,840	150	0.98	0.82	0.66	0.98	4,770	310	19,690	5,230	0.48	0.82	0.94	0.48	9,760	150	19,840	250	0.98	0.99	0.98	0.98
	Tymoviridae	9,830	1,100	18,900	170	0.98	0.96	0.90	0.98	7,610	410	19,590	2,390	0.76	0.91	0.95	0.76	9,680	1,370	18,630	320	0.97	0.94	0.88	0.97
	Unclassified	9,830	530	19,470	170	0.98	0.98	0.95	0.98	8,850	420	19,580	1,150	0.89	0.95	0.95	0.89	9,640	730	19,270	360	0.96	0.96	0.93	0.96
	Virgaviridae	9,850	220	19,760	150	0.99	0.99	0.98	0.99	9,420	430	19,560	570	0.94	0.97	0.96	0.94	9,630	430	19,560	360	0.96	0.97	0.96	0.96
Sugar beet	Alphaflexiviridae	9,550	1,580	18,410	440	0.96	0.93	0.86	0.96	8,410	380	19,610	1,580	0.84	0.93	0.96	0.84	9,700	360	19,620	300	0.97	0.98	0.96	0.97
	Alphasatellitidae	9,540	890	19,110	450	0.95	0.96	0.91	0.95	9,200	420	19,560	810	0.92	0.96	0.96	0.92	9,650	290	19,710	340	0.97	0.98	0.97	0.97
	Amalgaviridae	9,510	2,490	17,510	490	0.95	0.90	0.79	0.95	7,210	390	19,610	2,790	0.72	0.89	0.95	0.72	9,750	650	19,350	250	0.98	0.97	0.94	0.98
	Aspiviridae	9,560	6,480	13,520	440	0.96	0.77	0.60	0.96	3,570	330	19,670	6,430	0.36	0.77	0.92	0.36	9,730	330	19,670	270	0.97	0.98	0.97	0.97
	Benyviridae	9,580	720	19,280	420	0.96	0.96	0.93	0.96	8,810	370	19,630	1,190	0.88	0.95	0.96	0.88	9,710	810	19,190	290	0.97	0.96	0.92	0.97
	Betaflexiviridae	9,540	3,630	16,370	460	0.95	0.86	0.72	0.95	6,300	540	19,460	3,700	0.63	0.86	0.92	0.63	9,590	400	19,600	410	0.96	0.97	0.96	0.96

	Bromoviridae	9,570	300	19,710	430	0.96	0.98	0.97	0.96	9,620	330	19,680	380	0.96	0.98	0.97	0.96	9,730	460	19,540	280	0.97	0.98	0.95	0.97
	Caulimoviridae	9,530	4,950	15,050	470	0.95	0.82	0.66	0.95	4,280	430	19,570	5,720	0.43	0.80	0.91	0.43	9,680	1,130	18,870	320	0.97	0.95	0.90	0.97
	Closteroviridae	9,560	880	19,120	440	0.96	0.96	0.92	0.96	8,620	380	19,620	1,380	0.86	0.94	0.96	0.86	9,690	870	19,130	310	0.97	0.96	0.92	0.97
	Endornaviridae	9,540	670	19,330	470	0.95	0.96	0.93	0.95	8,990	370	19,650	1,000	0.90	0.95	0.96	0.90	9,730	710	19,290	280	0.97	0.97	0.93	0.97
	Fimoviridae	9,540	1,570	18,420	460	0.95	0.93	0.86	0.95	7,400	400	19,600	2,590	0.74	0.90	0.95	0.74	9,690	1,390	18,600	310	0.97	0.94	0.87	0.97
	Geminiviridae	9,530	1,360	18,650	460	0.95	0.94	0.88	0.95	8,760	480	19,510	1,250	0.88	0.94	0.95	0.88	9,580	290	19,710	420	0.96	0.98	0.97	0.96
	Genomoviridae	9,580	230	19,770	420	0.96	0.98	0.98	0.96	9,570	330	19,670	430	0.96	0.97	0.97	0.96	9,750	540	19,460	250	0.98	0.97	0.95	0.98
	Kitaviridae	9,520	290	19,710	480	0.95	0.97	0.97	0.95	9,540	440	19,560	460	0.95	0.97	0.96	0.95	9,670	540	19,460	330	0.97	0.97	0.95	0.97
	Mayoviridae	9,520	90	19,900	480	0.95	0.98	0.99	0.95	8,750	480	19,520	1,240	0.88	0.94	0.95	0.88	9,650	1,500	18,490	350	0.97	0.94	0.87	0.97
	Nanoviridae	9,600	320	19,670	390	0.96	0.98	0.97	0.96	9,670	260	19,720	330	0.97	0.98	0.97	0.97	9,730	400	19,590	260	0.97	0.98	0.96	0.97
	Partitiviridae	9,500	680	19,330	500	0.95	0.96	0.93	0.95	9,110	450	19,560	890	0.91	0.96	0.95	0.91	9,660	610	19,390	350	0.97	0.97	0.94	0.97
	Phenuiviridae	9,540	4,760	15,250	460	0.95	0.83	0.67	0.95	5,240	480	19,530	4,760	0.52	0.83	0.92	0.52	9,650	340	19,660	360	0.96	0.98	0.97	0.96
	Phycodnaviridae	9,560	1,400	18,600	440	0.96	0.94	0.87	0.96	6,390	320	19,680	3,610	0.64	0.87	0.95	0.64	9,750	2,580	17,420	250	0.98	0.91	0.79	0.98
	Potyviridae	9,550	1,860	18,130	450	0.96	0.92	0.84	0.96	7,880	350	19,650	2,110	0.79	0.92	0.96	0.79	9,730	620	19,370	270	0.97	0.97	0.94	0.97
	Reoviridae	9,610	650	19,350	390	0.96	0.97	0.94	0.96	5,170	270	19,730	4,830	0.52	0.83	0.95	0.52	9,750	4,550	15,450	250	0.98	0.84	0.68	0.98
	Rhabdoviridae	9,530	3,430	16,570	470	0.95	0.87	0.74	0.95	6,610	410	19,590	3,390	0.66	0.87	0.94	0.66	9,700	320	19,680	300	0.97	0.98	0.97	0.97
	Secoviridae	9,500	2,940	17,060	500	0.95	0.89	0.76	0.95	6,940	490	19,510	3,060	0.69	0.88	0.93	0.69	9,630	500	19,500	370	0.96	0.97	0.95	0.96
	Small families	9,550	540	19,450	450	0.96	0.97	0.95	0.96	8,140	380	19,620	1,850	0.81	0.93	0.96	0.81	9,720	1,660	18,330	280	0.97	0.94	0.85	0.97
	Solemoviridae	9,530	550	19,450	470	0.95	0.97	0.95	0.95	9,170	420	19,580	830	0.92	0.96	0.96	0.92	9,690	640	19,360	310	0.97	0.97	0.94	0.97
	Tolecusatellitidae	9,510	1,300	18,690	490	0.95	0.94	0.88	0.95	8,770	420	19,570	1,230	0.88	0.94	0.95	0.88	9,660	330	19,670	330	0.97	0.98	0.97	0.97
	Tombusviridae	9,590	210	19,770	410	0.96	0.98	0.98	0.96	9,730	340	19,650	260	0.97	0.98	0.97	0.97	9,730	380	19,610	260	0.97	0.98	0.96	0.97
	Tospoviridae	9,520	4,210	15,790	480	0.95	0.84	0.69	0.95	5,450	440	19,560	4,550	0.55	0.83	0.93	0.55	9,680	700	19,300	320	0.97	0.97	0.93	0.97
	Tymoviridae	9,530	620	19,380	470	0.95	0.96	0.94	0.95	7,370	470	19,530	2,630	0.74	0.90	0.94	0.74	9,640	2,370	17,630	360	0.96	0.91	0.80	0.96
	Unclassified	9,580	880	19,100	420	0.96	0.96	0.92	0.96	8,500	350	19,640	1,490	0.85	0.94	0.96	0.85	9,690	980	19,010	300	0.97	0.96	0.91	0.97
	Virgaviridae	9,560	490	19,520	440	0.96	0.97	0.95	0.96	9,280	450	19,560	720	0.93	0.96	0.95	0.93	9,640	590	19,410	370	0.96	0.97	0.94	0.96

Supplementary Table 4. tBLASTx results for the detection of novel viral fragments in the family leave-out setup. 10,000 random fragments of length 1000 were sampled from each family and aligned against the viral dataset by tBLASTx from which this family was removed. Shown are tBLASTx in terms of fraction of aligned fragments with no filters at all and after filtering.

Leave-out family	Fraction of aligned fragments	
	No filtering	percent identity > 0.5 & length > 50 aa & E-value < 10^{-10}
Alphaflexiviridae	1.000	0.907
Alphasatellitidae	1.000	1.000
Amalgaviridae	0.995	0.000
Aspiviridae	0.915	0.023
Benyviridae	0.994	0.261
Betaflexiviridae	0.999	0.785
Bromoviridae	0.994	0.654
Caulimoviridae	0.993	0.151
Closteroviridae	0.975	0.378
Endornaviridae	0.993	0.415
Fimoviridae	0.999	0.711
Geminiviridae	1.000	0.924
Genomoviridae	1.000	0.227
Kitaviridae	0.999	0.487
Mayoviridae	0.982	0.486
Nanoviridae	0.998	0.232
Partitiviridae	0.998	0.630
Phenuiviridae	0.978	0.302
Phycodnaviridae	0.983	0.004
Potyviridae	1.000	0.868
Reoviridae	0.844	0.000
Rhabdoviridae	0.998	0.235
Secoviridae	0.992	0.328
Small_families	0.992	0.368
Solemoviridae	1.000	0.930
Tolecusatellitidae	1.000	0.978
Tombusviridae	1.000	0.464
Tospoviridae	0.998	0.794
Tymoviridae	1.000	0.963
Unclassified	0.995	0.529
Virgaviridae	0.998	0.538

Supplementary Table 5. DeepVirFinder results for the detection of novel viral fragments in the family leave-out setup. Statistics were calculated based on prediction by DeepVirFinder of 20,000 random fragments of length 1000 sampled, 10,000 from the left aside viral family and 10,000 from the plant used for training. The column 'Fraction of bacterial fragments predicted as viral' contains prediction of DeepVirFinder for 10,000 fragments of length 1000 randomly sampled from bacteria.

Plant used for training	Leave-out family	TP	FP	TN	FN	TPR	Accuracy	Precision	Recall	Fraction of bacterial fragments predicted as viral
Peach	Alphaflexiviridae	6418	59	9941	3582	0.642	0.818	0.642	0.991	0.666
	Alphasatellitidae	6487	26	9974	3513	0.649	0.823	0.649	0.996	0.632
	Amalgaviridae	3076	10	9990	6924	0.308	0.653	0.308	0.997	0.589
	Aspiviridae	1987	11	9989	8013	0.199	0.599	0.199	0.994	0.619
	Benyviridae	8168	12	9988	1832	0.817	0.908	0.817	0.999	0.643
	Betaflexiviridae	3307	107	9893	6693	0.331	0.660	0.331	0.969	0.730
	Bromoviridae	9383	23	9977	617	0.938	0.968	0.938	0.998	0.589
	Caulimoviridae	2500	0	10000	7500	0.250	0.625	0.250	1.000	0.720
	Closteroviridae	8100	0	10000	1900	0.810	0.905	0.810	1.000	0.690
	Endornaviridae	6062	14	9986	3938	0.606	0.802	0.606	0.998	0.684
	Fimoviridae	6176	8	9992	3824	0.618	0.808	0.618	0.999	0.716
	Geminiviridae	5917	12	9988	4083	0.592	0.795	0.592	0.998	0.684
	Genomoviridae	8000	0	10000	2000	0.800	0.900	0.800	1.000	0.690
	Kitaviridae	9438	10	9990	562	0.944	0.971	0.944	0.999	0.684
	Mayoviridae	9526	39	9961	474	0.953	0.974	0.953	0.996	0.710
	Nanoviridae	8900	0	10000	1100	0.890	0.945	0.890	1.000	0.680
	Partitiviridae	8511	22	9978	1489	0.851	0.924	0.851	0.997	0.660
	Phenuiviridae	2800	0	10000	7200	0.280	0.640	0.280	1.000	0.800
	Phycodnaviridae	6058	55	9945	3942	0.606	0.800	0.606	0.991	0.638
	Potyviridae	7000	300	9700	3000	0.700	0.835	0.700	0.959	0.810
	Reoviridae	8440	26	9974	1560	0.844	0.921	0.844	0.997	0.698
	Rhabdoviridae	3363	18	9982	6637	0.336	0.667	0.336	0.995	0.685
	Secoviridae	3980	18	9982	6020	0.398	0.698	0.398	0.995	0.689
	Small families	8401	14	9986	1599	0.840	0.919	0.840	0.998	0.648
	Solemoviridae	6564	10	9990	3436	0.656	0.828	0.656	0.998	0.648
	Tolecusatellitidae	3800	0	10000	6200	0.380	0.690	0.380	1.000	0.680
	Tombusviridae	8595	11	9989	1405	0.860	0.929	0.860	0.999	0.703
	Tospoviridae	1721	28	9972	8279	0.172	0.585	0.172	0.984	0.722
	Tymoviridae	2622	26	9974	7378	0.262	0.630	0.262	0.990	0.541
	Unclassified	7300	0	10000	2700	0.730	0.865	0.730	1.000	0.650

	Virgaviridae	8459	43	9957	1541	0.846	0.921	0.846	0.995	0.710
Grapevine	Alphaflexiviridae	7634	73	9927	2366	0.763	0.878	0.763	0.991	0.837
	Alphasatellitidae	5808	40	9960	4192	0.581	0.788	0.581	0.993	0.803
	Amalgaviridae	6644	58	9942	3356	0.664	0.829	0.664	0.991	0.823
	Aspiviridae	3226	50	9950	6774	0.323	0.659	0.323	0.985	0.810
	Benyviridae	9252	34	9966	748	0.925	0.961	0.925	0.996	0.638
	Betaflexiviridae	3117	21	9979	6883	0.312	0.655	0.312	0.993	0.717
	Bromoviridae	9644	45	9955	356	0.964	0.980	0.964	0.995	0.804
	Caulimoviridae	2070	30	9970	7930	0.207	0.602	0.207	0.986	0.747
	Closteroviridae	9060	30	9970	940	0.906	0.952	0.906	0.997	0.744
	Endornaviridae	9146	28	9972	854	0.915	0.956	0.915	0.997	0.786
	Fimoviridae	4000	39	9961	6000	0.400	0.698	0.400	0.990	0.798
	Geminiviridae	7641	72	9928	2359	0.764	0.878	0.764	0.991	0.817
	Genomoviridae	8550	30	9970	1450	0.855	0.926	0.855	0.997	0.746
	Kitaviridae	9921	35	9965	79	0.992	0.994	0.992	0.996	0.790
	Mayoviridae	9366	36	9964	634	0.937	0.967	0.937	0.996	0.722
	Nanoviridae	9070	20	9980	930	0.907	0.953	0.907	0.998	0.772
	Partitiviridae	8847	33	9814	2320	0.792	0.888	0.792	0.996	0.724
	Phenuiviridae	3380	50	9950	6620	0.338	0.667	0.338	0.985	0.827
	Phycodnaviridae	7467	36	9964	2533	0.747	0.872	0.747	0.995	0.727
	Potyviridae	7433	40	9960	2567	0.743	0.870	0.743	0.995	0.786
	Reoviridae	8565	45	9955	1435	0.857	0.926	0.857	0.995	0.807
	Rhabdoviridae	3063	39	9961	6937	0.306	0.651	0.306	0.987	0.753
	Secoviridae	4256	22	9978	5744	0.426	0.712	0.426	0.995	0.774
	Small families	8787	66	9934	1213	0.879	0.936	0.879	0.993	0.829
	Solemoviridae	8000	54	9946	2000	0.800	0.897	0.800	0.993	0.806
	Tolecusatellitidae	5170	30	9970	4830	0.517	0.757	0.517	0.994	0.741
	Tombusviridae	8715	56	9944	1285	0.872	0.933	0.872	0.994	0.818
	Tospoviridae	1471	34	9966	8529	0.147	0.572	0.147	0.977	0.739
	Tymoviridae	3355	48	9952	6645	0.336	0.665	0.336	0.986	0.735
	Unclassified	7850	40	9960	2150	0.785	0.891	0.785	0.995	0.812
	Virgaviridae	9308	60	9940	692	0.931	0.962	0.931	0.994	0.803
Sugar beet	Alphaflexiviridae	4975	95	9905	5025	0.498	0.744	0.498	0.981	0.225
	Alphasatellitidae	6923	148	9852	3077	0.692	0.839	0.692	0.979	0.427
	Amalgaviridae	2784	159	9841	7216	0.278	0.631	0.278	0.946	0.382
	Aspiviridae	2491	113	9887	7509	0.249	0.619	0.249	0.957	0.338
	Benyviridae	7156	171	9829	2844	0.716	0.849	0.716	0.977	0.366
	Betaflexiviridae	4630	2020	7980	5370	0.463	0.631	0.463	0.696	0.415
	Bromoviridae	9487	464	9536	513	0.949	0.951	0.949	0.953	0.772

	Caulimoviridae	1460	130	9870	8540	0.146	0.567	0.146	0.918	0.315
	Closteroviridae	7870	90	9910	2130	0.787	0.889	0.787	0.989	0.274
	Endornaviridae	8184	183	9817	1816	0.818	0.900	0.818	0.978	0.439
	Fimoviridae	2412	212	9788	7588	0.241	0.610	0.241	0.919	0.441
	Geminiviridae	7152	210	9790	2848	0.715	0.847	0.715	0.971	0.432
	Genomoviridae	9090	180	9820	910	0.909	0.946	0.909	0.981	0.336
	Kitaviridae	8588	82	9918	1412	0.859	0.925	0.859	0.991	0.235
	Mayoviridae	8072	67	9933	1928	0.807	0.900	0.807	0.992	0.203
	Nanoviridae	5780	210	9790	4220	0.578	0.779	0.578	0.965	0.395
	Partitiviridae	8065	161	9839	1935	0.807	0.895	0.807	0.980	0.400
	Phenuiviridae	1890	120	9880	8110	0.189	0.589	0.189	0.940	0.312
	Phycodnaviridae	6479	161	9839	3521	0.648	0.816	0.648	0.976	0.482
	Potyviridae	6770	220	9780	3230	0.677	0.828	0.677	0.969	0.432
	Reoviridae	6309	101	9899	3691	0.631	0.810	0.631	0.984	0.271
	Rhabdoviridae	4267	94	9906	5733	0.427	0.709	0.427	0.978	0.326
	Secoviridae	3457	93	9907	6543	0.346	0.668	0.346	0.974	0.278
	Small families	8057	128	9872	1943	0.806	0.896	0.806	0.984	0.373
	Solemoviridae	7347	85	9915	2653	0.735	0.863	0.735	0.989	0.309
	Tolecusatellitidae	4980	140	9860	5020	0.498	0.742	0.498	0.973	0.299
	Tombusviridae	8321	122	9878	1679	0.832	0.910	0.832	0.986	0.369
	Tospoviridae	3345	89	9911	6655	0.335	0.663	0.335	0.974	0.265
	Tymoviridae	4585	97	9903	5415	0.459	0.724	0.459	0.979	0.269
	Unclassified	7455	164	9836	2545	0.746	0.865	0.746	0.978	0.353
	Virgaviridae	8172	107	9893	1828	0.817	0.903	0.817	0.987	0.361

Supplementary Table 6. VirSorter2 results for the detection of novel viral fragments in the family leave-out setup. Statistics were calculated based on prediction by VirSorter2 of 20,000 random fragments of length 1000 sampled, 10,000 from the viral family and 10,000 from the peach genome.

Leave-out family	TP	FP	TN	FN	TPR	Accuracy	Precision	Recall
Alphaflexiviridae	1820	20	9980	8180	0.182	0.590	0.989	0.182
Alphasatellitidae	9908	20	9980	92	0.991	0.994	0.998	0.991
Amalgaviridae	6116	20	9980	3884	0.612	0.805	0.997	0.612
Aspiviridae	345	20	9980	9655	0.035	0.516	0.945	0.035
Benyviridae	689	20	9980	9311	0.069	0.533	0.972	0.069
Betaflexiviridae	1611	20	9980	8389	0.161	0.580	0.988	0.161
Bromoviridae	2214	20	9980	7786	0.221	0.610	0.991	0.221
Caulimoviridae	718	20	9980	9282	0.072	0.535	0.973	0.072
Closteroviridae	977	20	9980	9023	0.098	0.548	0.980	0.098
Endornaviridae	0	20	9980	10000	0.000	0.499	0.000	0.000
Fimoviridae	420	20	9980	9580	0.042	0.520	0.955	0.042
Geminiviridae	3256	20	9980	6744	0.326	0.662	0.994	0.326
Genomoviridae	4251	20	9980	5749	0.425	0.712	0.995	0.425
Kitaviridae	1061	20	9980	8939	0.106	0.552	0.981	0.106
Mayoviridae	2669	20	9980	7331	0.267	0.632	0.993	0.267
Nanoviridae	4485	20	9980	5515	0.449	0.723	0.996	0.449
Partitiviridae	4843	20	9980	5157	0.484	0.741	0.996	0.484
Phenuiviridae	452	20	9980	9548	0.045	0.522	0.958	0.045
Phycodnaviridae	3218	20	9980	6782	0.322	0.660	0.994	0.322
Potyviridae	1659	20	9980	8341	0.166	0.582	0.988	0.166
Reoviridae	513	20	9980	9487	0.051	0.525	0.962	0.051
Rhabdoviridae	1064	20	9980	8936	0.106	0.552	0.982	0.106
Secoviridae	1049	20	9980	8951	0.105	0.551	0.981	0.105
Small families	1686	20	9980	8314	0.169	0.583	0.988	0.169
Solemoviridae	4174	20	9980	5826	0.417	0.708	0.995	0.417
Tolecusatellitidae	5	20	9980	9995	0.001	0.499	0.200	0.001
Tombusviridae	2934	20	9980	7066	0.293	0.646	0.993	0.293
Tospoviridae	413	20	9980	9587	0.041	0.520	0.954	0.041
Tymoviridae	2740	20	9980	7260	0.274	0.636	0.993	0.274
Unclassified	2552	20	9980	7448	0.255	0.627	0.992	0.255
Virgaviridae	2443	20	9980	7557	0.244	0.621	0.992	0.244

Supplementary Table 7. VirHunter model trained on rice dataset is used to predict classes of fragments from 6 test datasets: 4 plants (peach, grapevine, sugar beet and rice), viruses and bacteria. VirHunter model is trained following the procedure in section 2.6 with rice used as plant genome. Shown are classification results of this model when applied to test datasets built by randomly drawing 10,000 fragments of length 1000 bp from peach, sugar beet, grapevine, rice as well as from all viral sequences and from bacteria. Predictions for the expected class are shown in bold.

Plant used for training	Test dataset	Predicted label		
		Plant	Virus	Bacteria
Rice	peach	**0.766**	0.171	0.064
	grapevine	**0.759**	0.151	0.09
	sugar beet	**0.702**	0.192	0.107
	rice	**0.984**	0.012	0.003
	virus	0.003	**0.996**	0.002
	bacteria	0.006	0.018	**0.977**

Supplementary Table 8. Statistics of performance of VirHunter, DeepVirfinder and VirSorter2 on the 12 RNAseq virome data. The statistics were calculated from the results in Table 2. Prediction time was measured with one CPU.

Tool	Dataset and host		TP	FP	TN	FN	Accuracy	Precision	Recall	Prediction time, seconds
VirHunter	P1	Peach	2	33	974	0	0.967	0.057	1.000	116
	P2	Peach	2	17	396	0	0.959	0.105	1.000	125
	P3	Peach	2	21	662	0	0.969	0.087	1.000	112
	G1	grapevine	10	143	9001	0	0.984	0.065	1.000	158
	G2	grapevine	10	168	16846	0	0.990	0.056	1.000	282
	G3	grapevine	18	190	18540	2	0.990	0.087	0.900	355
	G4	grapevine	14	81	4236	1	0.981	0.147	0.933	379
	G5	grapevine	23	239	19131	2	0.988	0.088	0.920	160
	G6	grapevine	14	56	2861	1	0.981	0.200	0.933	416
	S1	sugar beet	10	226	5845	1	0.963	0.042	0.909	227
	S2	sugar beet	16	261	8625	0	0.971	0.058	1.000	276
	S3	sugar beet	11	192	6709	0	0.972	0.054	1.000	251
DeepVirFinder	P1	Peach	2	43	964	0	0.957	0.044	1.000	2332
	P2	Peach	2	30	383	0	0.928	0.063	1.000	1412
	P3	Peach	2	47	636	0	0.931	0.041	1.000	1794
	G1	grapevine	6	127	9017	4	0.986	0.045	0.600	11688
	G2	grapevine	9	122	16892	1	0.993	0.069	0.900	16107
	G3	grapevine	17	120	18610	3	0.993	0.124	0.850	18509
	G4	grapevine	11	70	4247	4	0.983	0.136	0.733	7841
	G5	grapevine	23	279	19091	2	0.986	0.076	0.920	20460
	G6	grapevine	13	73	2844	2	0.974	0.151	0.867	5597
	S1	sugar beet	11	324	5747	0	0.947	0.033	1.000	14326
	S2	sugar beet	16	403	8483	0	0.955	0.038	1.000	21335
	S3	sugar beet	11	296	6605	0	0.957	0.036	1.000	15377
VirSorter2	P1	Peach	1	9	999	1	0.990	0.100	0.500	1576
	P2	Peach	1	7	407	1	0.981	0.125	0.500	1200
	P3	Peach	1	6	678	1	0.990	0.143	0.500	1624
	G1	grapevine	4	48	9102	6	0.994	0.077	0.400	10389

	G2	grapevine	6	111	16907	4	0.993	0.051	0.600	18896
	G3	grapevine	11	131	18608	9	0.993	0.077	0.550	20401
	G4	grapevine	4	20	4308	11	0.993	0.167	0.267	5681
	G5	grapevine	8	136	19251	17	0.992	0.056	0.320	2396
	G6	grapevine	12	14	2906	3	0.994	0.462	0.800	4497
	S1	sugar beet	6	22	6054	5	0.996	0.214	0.545	6724
	S2	sugar beet	7	30	8865	9	0.996	0.189	0.438	8857
	S3	sugar beet	4	17	6891	7	0.997	0.190	0.364	7401

Supplementary Table 9. VirHunter's individual neural networks do not show signs of overfitting. Shown is the accuracy of individual networks trained for leave-out datasets of peach following the procedure described in Section 2.6.

Plant used for training	Leave-out family	Individual networks with different k								
		k=5			k=7			k=10		
		train accuracy	validation accuracy	test accuracy	train accuracy	validation accuracy	test accuracy	train accuracy	validation accuracy	test accuracy
Peach	Alphaflexiviridae	0.942	0.949	0.955	0.965	0.970	0.975	0.984	0.985	0.987
	Alphasatellitidae	0.943	0.952	0.959	0.964	0.971	0.973	0.990	0.988	0.990
	Amalgaviridae	0.943	0.951	0.958	0.965	0.969	0.977	0.984	0.984	0.988
	Aspiviridae	0.943	0.953	0.958	0.965	0.971	0.970	0.988	0.988	0.992
	Benyviridae	0.942	0.950	0.956	0.964	0.970	0.978	0.984	0.984	0.987
	Betaflexiviridae	0.948	0.956	0.960	0.967	0.973	0.976	0.986	0.987	0.989
	Bromoviridae	0.943	0.952	0.957	0.965	0.971	0.975	0.985	0.985	0.987
	Caulimoviridae	0.944	0.954	0.958	0.966	0.972	0.975	0.985	0.984	0.989
	Closteroviridae	0.943	0.951	0.962	0.965	0.969	0.974	0.990	0.988	0.989
	Endornaviridae	0.943	0.950	0.953	0.964	0.970	0.975	0.990	0.988	0.989
	Fimoviridae	0.944	0.951	0.957	0.965	0.971	0.974	0.985	0.985	0.988
	Geminiviridae	0.940	0.948	0.952	0.963	0.967	0.974	0.984	0.984	0.986
	Genomoviridae	0.943	0.952	0.956	0.964	0.970	0.975	0.985	0.986	0.988
	Kitaviridae	0.942	0.952	0.959	0.964	0.969	0.977	0.990	0.987	0.990
	Mayoviridae	0.943	0.953	0.956	0.964	0.971	0.975	0.984	0.985	0.987
	Nanoviridae	0.953	0.959	0.956	0.969	0.974	0.975	0.986	0.987	0.988
	Partitiviridae	0.944	0.951	0.959	0.964	0.970	0.975	0.984	0.984	0.986
	Phenuiviridae	0.944	0.951	0.958	0.964	0.971	0.975	0.989	0.988	0.990
	Phycodnaviridae	0.945	0.955	0.958	0.968	0.972	0.977	0.987	0.987	0.988
	Potyviridae	0.940	0.948	0.955	0.962	0.966	0.972	0.983	0.980	0.989
	Reoviridae	0.943	0.951	0.959	0.964	0.969	0.973	0.985	0.985	0.988
	Rhabdoviridae	0.946	0.955	0.961	0.965	0.971	0.970	0.985	0.986	0.987
	Secoviridae	0.947	0.955	0.961	0.967	0.971	0.978	0.986	0.986	0.989
	Small families	0.943	0.952	0.957	0.965	0.971	0.975	0.984	0.983	0.990
	Solemoviridae	0.943	0.950	0.959	0.965	0.970	0.977	0.985	0.986	0.986
	Tolecusatellitidae	0.942	0.952	0.959	0.963	0.969	0.972	0.984	0.984	0.987
	Tombusviridae	0.942	0.951	0.957	0.965	0.972	0.975	0.989	0.988	0.990
	Tospoviridae	0.944	0.952	0.958	0.965	0.970	0.975	0.984	0.986	0.988
	Tymoviridae	0.943	0.950	0.956	0.965	0.971	0.975	0.991	0.998	0.990
	Unclassified	0.944	0.952	0.957	0.966	0.971	0.973	0.985	0.986	0.987
	Virgaviridae	0.943	0.951	0.959	0.964	0.968	0.973	0.985	0.986	0.987
